# Comparative Evaluation of Thiol- and Amine-Conjugating Moieties for Endogenous Albumin Binding after Intravenous Administration

**DOI:** 10.1021/acsptsci.5c00240

**Published:** 2025-06-26

**Authors:** Anja Federa, Hemma Schueffl, Iris K. Minichmayr, Alexander Kastner, Julia Kronberger, Thomas L. Mindt, Petra Heffeter, Christian R. Kowol

**Affiliations:** † Institute of Inorganic Chemistry, Faculty of Chemistry, 27258 University of Vienna, Waehringer Str. 42, A-1090 Vienna, Austria; ‡ Vienna Doctoral School in Chemistry, University of Vienna, Waehringer Str. 42, 1090 Vienna, Austria; § Center for Cancer Research and Comprehensive Cancer Center, 27271 Medical University of Vienna, Borschkegasse 8a, A-1090 Vienna, Austria; ∥ Department of Clinical Pharmacology, Medical University of Vienna, Waehringer Guertel 18-20, A-1090 Vienna, Austria; ⊥ Joint Applied Medicinal Radiochemistry Facility of the University of Vienna and the Medical University Vienna, 1010 Vienna, Austria; # Research Cluster “Translational Cancer Therapy Research”, 1090 Vienna, Austria

**Keywords:** albumin, platinum, maleimide, drug delivery, oxadiazole, anticancer

## Abstract

Maleimides are widely used in anticancer drug development for linking small-molecule drugs to macromolecules like antibodies or proteins via thiol-Michael addition reactions. Despite their widespread use, even in clinically approved therapeutics, they present significant drawbacks such as hydrolysis at physiological pH and instability of the formed thiosuccinimide bond. Hence, there is a growing need for more stable yet equally efficient binding units. This is particularly important for drug-delivery systems that bind to endogenous albumin in vivo, exploiting the ability of the protein to accumulate in tumor tissue. This study compares phenyloxadiazolyl methyl sulfone (PODS) and a 2,4-difluorophenyl sulfonamide (DFSA) derivative with maleimide as endogenous albumin binders. Of note, PODS and maleimide bind to Cys34, whereas DFSA targets Lys64 of albumin. The albumin binders were conjugated as axial ligands to oxaliplatin­(IV) complexes (**PODS-Ox-OAc** and **DFSA-Ox-OAc**) and studied in comparison to a maleimide-bearing reference compound (**Mal-Ox-OAc**). Both PODS- and DFSA-complexes showed higher hydrolytic stability at pH 7.4 than the maleimide complex. Albumin-binding was highly efficient for the PODS and maleimide complexes. However, the DFSA derivative exhibited only slow conjugation. This was also reflected in the serum pharmacokinetic and organ distribution studies using CT26 colon cancer-bearing mice. Here, the PODS complex showed the highest platinum levels in both serum and tumor tissue. Additionally, **PODS-Ox-OAc** induced the most significant tumor regression and prolonged overall survival in this model. Together, our data highlight PODS as a promising alternative to maleimide as an endogenous albumin binder.

## Graphical abstract

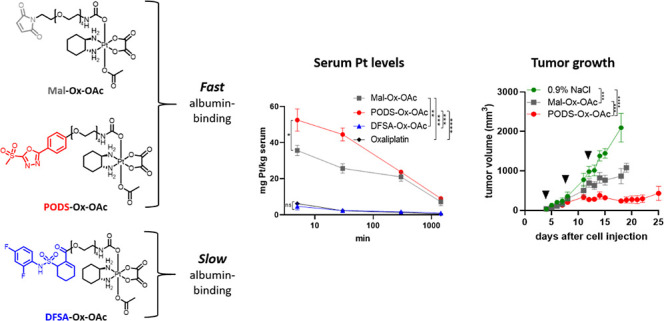

The treatment of cancer remains one of the most intricate challenges to be solved by modern medicine. A major hurdle in anticancer drug development is the inability of most treatments to differentiate effectively between cancerous and healthy cells, resulting in collateral damage also to normal tissues.^1,2^ Therefore, anticancer therapy requires a compromise between tolerable toxicity for the patient and sufficient damage to tumor cells.^3^ This so-called therapeutic index is generally narrow for anticancer drugs and their full potential is rarely exploited due to dose-limiting toxicities.^4^ As such, increasing focus is placed on broadening that window by converting conventional drugs into prodrugs and/or incorporating them into drug-delivery systems.^5,6^ Generally, the former entails chemical derivatization to improve tumor-specific activation in cancerous tissue, whereas the latter implies the modification of a drug to convey it to a specific target site in the body, where it is released from the carrier molecule.^7^ This in turn requires the delivery vehicle to accumulate in malignant rather than in healthy tissues. In cancer therapy, this is usually achieved by using macromolecules like nanoparticles, monoclonal antibodies (mAbs) or proteins.^8^ According to the enhanced permeability and retention (EPR) effect, macromolecules can passively enter the tumor tissue because of fenestrations in the vascularization network caused by abnormal vessel growth, and are retained there due to impaired lymphatic drainage. Notably, vehicles like mAbs or proteins can also target cancer-associated, overexpressed cell–surface receptors, adding an active tumor-targeting effect.^9^

Human serum albumin (HSA) is a promising drug carrier, especially due to its unique pharmacokinetic profile. With concentrations of about 35–50 g/L, HSA is the most abundant serum protein. Moreover, it has a circulatory half-life of ∼19 days and is known to accumulate both actively and passively in tumor tissue.^10–12^ As such, Abraxane, an albumin nanoparticle formulation of electrostatically bound paclitaxel, became the first approved chemotherapeutic agent utilizing albumin-mediated drug delivery in 2005.^10^ Interestingly, a different approach to achieve albumin conjugation was developed by Kratz et al. with the synthesis of a maleimide derivative of doxorubicin (Aldoxorubicin). Upon intravenous administration of the small molecule, the maleimide binds to the Cys34 thiol moiety of endogenous HSA in the patient'´s body, which makes up most of the thiol content in human blood.^13,14^ This approach is unique, since therapeutic bioconjugates are usually synthesized ex vivo prior to their application. Notably, Aldoxorubicin has completed a phase III clinical trial (http://www.clinicaltrials.gov; NCT02049905) for the treatment of soft tissue sarcoma (STS) in patients who had relapsed or not responded well to preceding chemotherapy, and performed considerably superior to standard treatments.^15^ Yet, despite conceptual clinical success and the maleimide’s monopolistic status as a rapid, site-specific, selective and highly efficient thiol-Michael addition reagent, maleimides entail two significant drawbacks: (1) they are susceptible to (fast) hydrolysis at neutral to basic pH values and (2) the thiosuccinimide bond in the final conjugates is prone to retro-Michael and thiol exchange reactions, causing loss of payload from the macromolecular carrier in vivo.^16^ Hence, an alternative to maleimide-mediated albumin conjugation is needed, which is equally reactive for in vivo conjugation, but more stable.

Although there exists a variety of electrophiles with thiol-binding properties for bioconjugation purposes (e.g., mono- and dibromomaleimides, iodoacetamides, vinyl sulfones, disulfides and diselenides, alkynones^17^), to our knowledge none of them has been investigated for the application as an endogenous binder after intravenous administration. After thorough literature research, two compounds previously published by Barbas et al. were selected for investigation: phenyloxadiazolyl methyl sulfone (PODS) and a 2,4-difluorophenyl sulfonamide (DFSA) derivative of the toll-like receptor 4 (TLR4) inhibitor Resatorvid (TAK-242). In 2013, PODS was reported to selectively and quantitatively react with thiols at pH 7.4 within only 5 min, while no reaction products with O- and N-substituents were observed. In addition, protein conjugates with PODS were significantly more stable and did not undergo thiol exchange reactions compared to thiosuccinimide linkages.^18^ Several groups have since explored PODS vs maleimide conjugation in different applications like small molecule-protein conjugation^19,20^ and radiolabeling of peptides^21^ or antibodies.^22^ However, its endogenous albumin-binding properties have not been reported so far.

The DFSA strategy is based on specific targeting of lysine in albumin. Usually, protein *N*-functionalization comes at the cost of site selectivity and poorly controllable substitution numbers, due to the high lysine abundance and their varying reactivity in proteins.^23^ However, a study by Barbas et al. from 2014 showed that DFSA selectively binds to Lys64 of HSA. In addition to site-specific albumin binding, the authors reported that no side reactions with other serum proteins occurred. TLR4 affinity was lacking due to polyethylene glycol (PEG) modification of the ester moiety and the stability of the final HSA-conjugate was largely increased in serum in vivo compared to maleimide.^24^ Mechanistically, the conjugation of albumin to PODS and DFSA proceeds differently than the Michael addition reaction to maleimides. For DFSA, the addition of a nucleophile to the cyclohexene-double bond is followed by allylic rearrangement and, consequently, elimination of the sulfonamide moiety.^24^ In case of PODS-like structures, a nucleophilic aromatic substitution takes place with the methylsulfone as the leaving group.^25,26^

This study aimed to investigate the potential of PODS and DFSA as alternatives to maleimide for the use as binders to endogenous albumin. In the past, we transferred the in vivo binding concept to metal-based anticancer agents by introducing maleimides as axial ligands of oxaliplatin­(IV) prodrugs, which yielded highly superior anticancer activity in murine in vivo models compared to clinically approved oxaliplatin.^27,28^ Therefore, we herein report the synthesis of PODS, DFSA and maleimide ligands and their subsequent complexation with oxaliplatin­(IV) for comparative evaluation. The final compounds were analyzed for their albumin-binding properties and kinetics in serum. Additionally, we show the crucial impact of rapid and stable albumin-drug conjugate formation on tissue distribution, tumor accumulation and anticancer activity in mice.

## Results and Discussion

### Synthesis, Stability and Reduction Properties

The synthesis of PODS-, DFSA- and maleimide-bearing oxaliplatin­(IV) complexes (**PODS-Ox-OAc**, **DFSA-Ox-OAc** and **Mal-Ox-OAc**) was performed via two methods in which the respective albumin-binding units were introduced to an oxaliplatin­(IV) precursor (Scheme 1). In a first step, oxaliplatin was oxidized asymmetrically using H_2_O_2_ in acetic acid to give the platinum­(IV) complex OxOAcOH with an acetato and hydroxido ligand in axial positions.^29^ Literature procedures were used to synthesize PODS^30^ and DFSA,^24,31^ which were subsequently functionalized with a PEG_4_-chain using a Mitsunobu reaction with commercially available HO-PEG_4_-NHBoc to guarantee sufficient aqueous solubility of the target compounds. Subsequently, the Boc-amine was deprotected with trifluoroacetic acid (TFA). For the preparation of **PODS-Ox-OAc** and **DFSA-Ox-OAc**, a method from Gibson et al. was employed using *N*,*N*′-disuccinimidylcarbonate (DSC) to generate a platinum­(IV) *N*-hydroxysuccinimide (NHS) active ester, followed by nucleophilic substitution with the amine to yield an axial carbamate moiety.^32^ Initial attempts at one-pot coupling reactions of the amines to the platinum­(IV) precursor in *N*,*N′*-dimethylformamide (DMF), in which OxOAcOH was activated in situ with DSC, only gave poor yields of 26% **PODS-Ox-OAc** and 11% **DFSA-Ox-OAc**. Therefore, the NHS active ester was isolated by precipitation with Et_2_O, giving OxOAcNHS in excellent yield. By combining PODS-PEG_4_-NH_2_ or DFSA-PEG_4_-NH_2_ with purified OxOAcNHS (Scheme 1), yields were increased to 41% **PODS-Ox-OAc** and 19% **DFSA-Ox-OAc**, respectively.

**Scheme 1:**
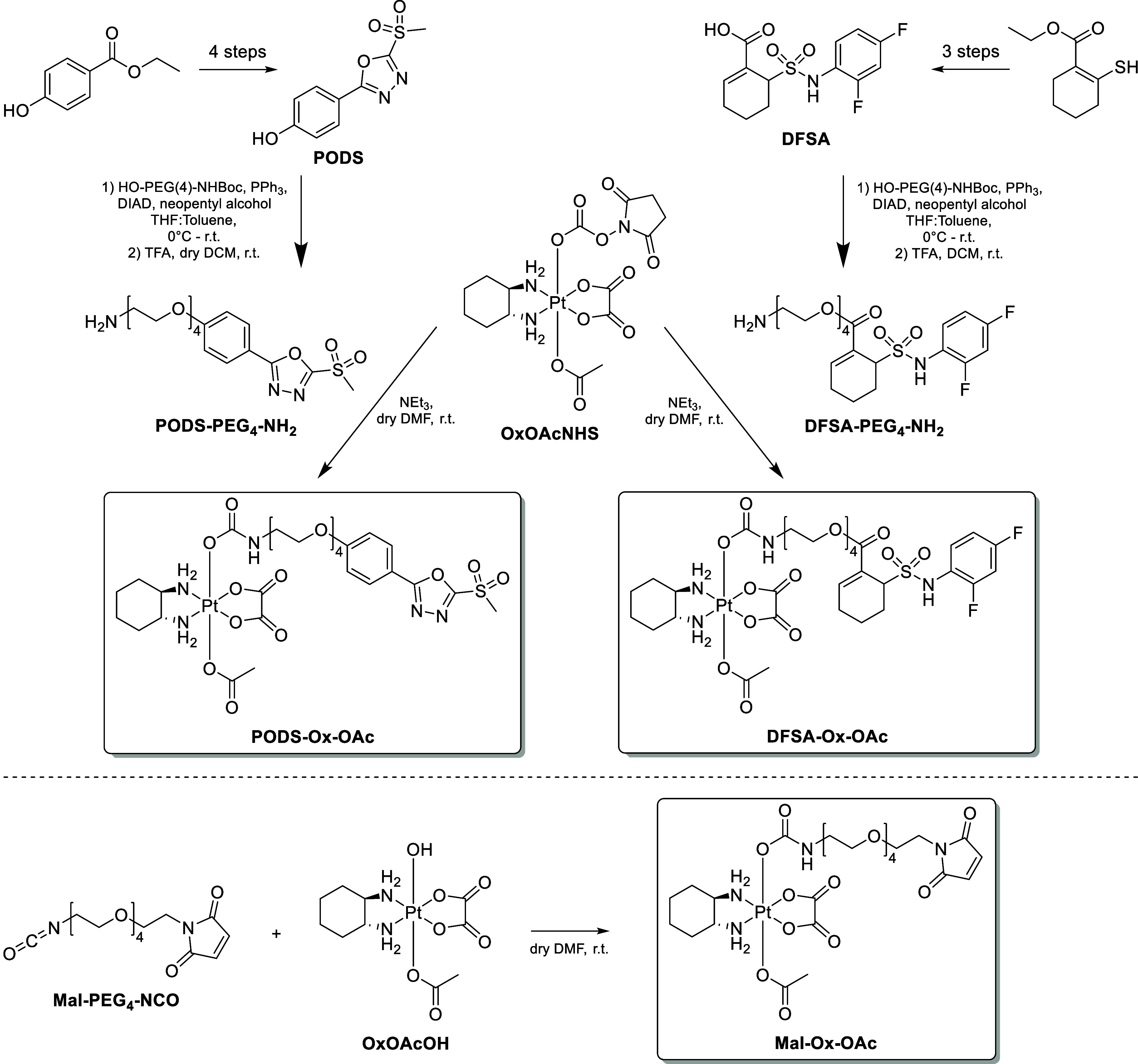
Synthesis Routes for **PODS-Ox-OAc**, **DFSA-Ox-OAc** and **Mal-Ox-OAc** ^a^

a Each platinum­(IV) complex bears a different thiol- or amine-conjugating moiety. The precursors PODS,^30^ DFSA,^24,31^ Mal-PEG_4_-NCO^33^ and OxOAcOH^29^ were synthesized from literature procedures.

A different method was used for the preparation of **Mal-Ox-OAc**, in which the ligand was directly coupled to OxOAcOH as an isocyanate (Scheme 1). This was necessary as maleimides undergo aza-Michael additions in the presence of amines, which would cause a significant amount of side reactions. Hence, Mal-PEG_4_-NCO was synthesized according to literature procedures^33^ from commercially available Mal-PEG_4_-COOH. NaN_3_ and ethyl chloroformate were used to generate the acyl azide intermediate, followed by a Curtius rearrangement. The obtained isocyanate was then reacted with OxOAcOH in DMF to give **Mal-Ox-OAc** in 22% yield. All final complexes were characterized by NMR spectroscopy, high-resolution mass spectrometry (HRMS), analytical HPLC and elemental analysis.

To determine whether PODS and DFSA offer a better alternative to maleimide, first the hydrolytic stability of **PODS-Ox-OAc** and **DFSA-Ox-OAc** in reference to **Mal-Ox-OAc** was investigated. Therefore, the compounds were incubated at 0.5 mM in 100 mM phosphate buffer (PB) at pH 7.4 and 20 °C and measured via HPLC (Figure 1). After 25 h only 23% of **Mal-Ox-OAc** were intact, whereas 70% **PODS-Ox-OAc** and 97% **DFSA-Ox-OAc** were detected, indicating a much higher hydrolytic stability compared to maleimide. In order to confirm that the decline of **Mal-Ox-OAc** and **PODS-Ox-OAc** can be attributed to ligand hydrolysis, the samples were additionally analyzed via HPLC-MS after ∼27 h. Indeed, the mass spectra of the newly formed peaks correspond to the hydrolysis products of the respective albumin-binding units: maleamic acid and oxadiazol-2-ol (Figure S1). Furthermore, all compounds (0.5 mM solutions) were investigated in different buffer systems and pH values at 20 °C, namely 100 mM PB at pH 5.5 and 6.5 (Figure S2A,B), as well as 20 mM HEPES (2-[4-(2-hydroxyethyl)­piperazin-1-yl]­ethane-1-sulfonic acid) and MOPS (3-(morpholin-4-yl)­propane-1-sulfonic acid) at pH 7.4 (Figure S2C,D). The relative peak areas of the hydrolyzed and intact compound peaks were compared and are listed in Table S1. As expected, the maleimide hydrolysis of **Mal-Ox-OAc** was substantially slower in acidic conditions with 80% of intact compound at pH 6.5 and even 96% at pH 5.5 after 25 h of incubation. Similarly, 94% and 99% of **PODS-Ox-OAc** remained intact at pH 6.5 and 5.5, respectively. Notably, when **Mal-Ox-OAc** was incubated in HEPES and MOPS buffers at pH 7.4, ∼60% of the complex remained intact, distinctly more than the 23% observed in PB, which is likely due to the lower buffer concentration. Surprisingly, for **PODS-Ox-OAc** in HEPES buffer an additional peak, apart from the intact compound and hydrolysis product, was observed (Figure S2C), which was identified as the HEPES substitution product via HPLC-MS (Figure S3), indicating that in HEPES the aliphatic OH substitutes the PODS-methylsulfone group. This was further supported by the fact that in MOPS buffer, missing this aliphatic OH group, no such conjugate was detected. **DFSA-Ox-OAc** was stable in all tested conditions. Conclusively, also in the other two buffers at pH 7.4 **Mal-Ox-OAc** hydrolyzed distinctly faster than **PODS-Ox-OAc**.

**Figure 1:**
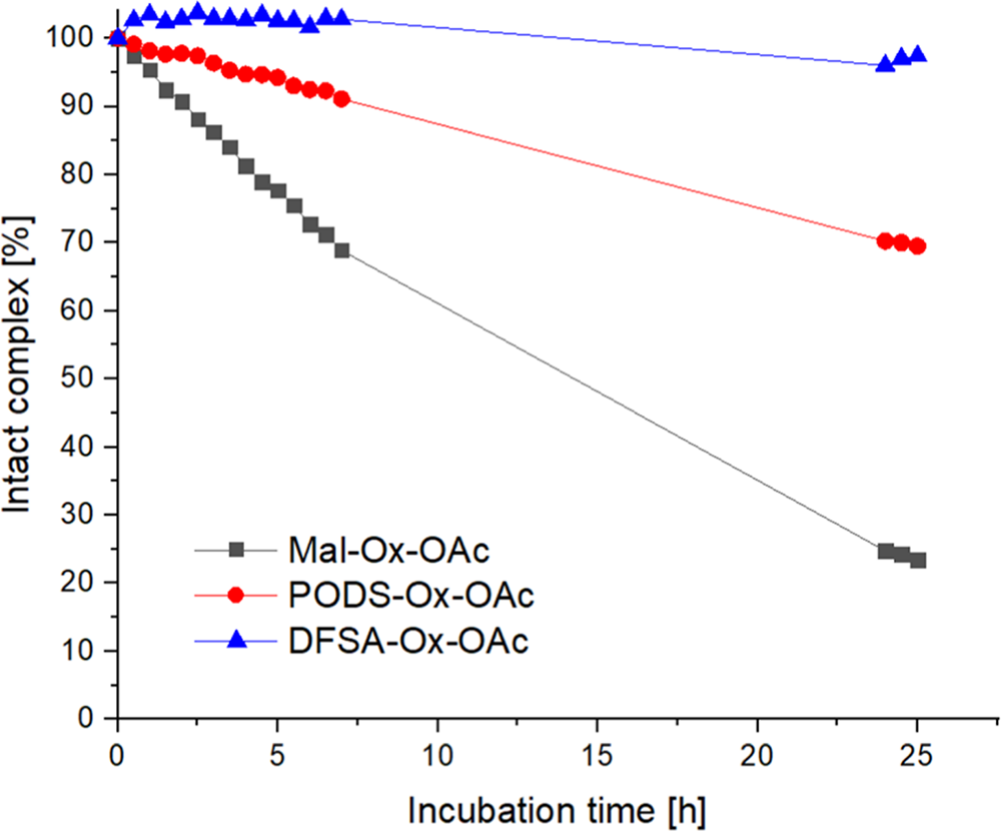
Hydrolytic stability of 0.5 mM **Mal-Ox-OAc**, **PODS-Ox-OAc** and **DFSA-Ox-OAc** in 100 mM PB at pH 7.4 and 20 °C over 25 h. All compound peaks were integrated at λ = 220 nm.

As a crucial step in the activation of platinum­(IV) complexes, the reduction of all three complexes to platinum­(II) was analyzed via HPLC at a concentration of 0.5 mM in 150 mM PB at pH 7.4 and 20 °C over 25 h in the presence of 30 eq. ascorbic acid (AA; Figure S4A). Due to the simultaneously occurring ligand hydrolysis (see above), the increase of released oxaliplatin was quantified (Figure S4B) rather than the decline of the parental compound peak. For **Mal-Ox-OAc,** 18% oxaliplatin were detected after 25 h, whereas for **PODS-Ox-OAc** 9% and for **DFSA-Ox-OAc** 10% were observed, unexpectedly revealing a ∼2-fold faster reduction rate for **Mal-Ox-OAc**. Of note, oxaliplatin itself exhibited ∼15% hydrolysis after 25 h at pH 7.4 and 20 °C, indicating that this side reaction did not distinctly affect the reduction measurements (Table S2).

### SEC-ICP-MS Measurements

To confirm in situ-binding to endogenous albumin, it was necessary to verify that, like maleimide, PODS and DFSA bind rapidly and efficiently to albumin. Therefore, we incubated the three complexes at 100 μM in mouse serum buffered with 150 mM PB at 37 °C and analyzed the samples over 24 h with size-exclusion chromatography coupled to inductively coupled plasma mass spectrometry (SEC-ICP-MS) (Figure 2A–C). Both ^195^Pt and ^48^SO contents were measured. The ^48^SO-trace shows the sulfur of serum proteins which are separated by their molecular weight into the high-molecular weight fraction (HMWF; ∼0–5 min) and the low-molecular-weight fraction (LMWF; >5 min) with albumin eluting at ∼4.0 min. Peaks in the ^195^Pt trace that elute coincidentally with the sulfur trace indicate whether the respective platinum-containing species are protein-bound or not. About 35% of **Mal-Ox-OAc** were bound to albumin at the 0 h time point (immediately after sample preparation), increasing to 75% after 1 h (Figure 2A and Table S3). Similarly, **PODS-Ox-OAc** was 30% albumin-bound at 0 h and reached a maximum binding of ∼80% after 1 h. Both albumin-conjugates remained stable over 24 h. In contrast, virtually no **DFSA-Ox-OAc** was albumin-bound at 0 h, increasing to ∼65% after 1 h and maximum binding of ∼80% only after 2 h. Interestingly, the conjugate did not remain fully stable, as evidenced by a decrease in the HMWF to 70% after 24 h. To gain a better understanding of the binding kinetics, additional experiments with 10 min intervals for 1 h were conducted (Figure 2D–F and Table S3). After 10 min, ∼60% of **Mal-Ox-OAc** and **PODS-Ox-OAc**, but only 18% of **DFSA-Ox-OAc** were bound to albumin. At this time point a new signal in the LMWF was observed for **Mal-Ox-OAc** (Figure S5), most likely originating from the maleimide hydrolysis. Since DFSA was specifically developed as a binder for HSA,^24^ we also investigated the binding properties of the resulting complexes in human serum (Figure S6 and Table S4). Unexpectedly, the binding for **DFSA-Ox-OAc** was even slower with 34% albumin-bound after 1 h and a maximum of 66% after 5 h. After 24 h, a decline to 59% platinum in the HMWF was observed, indicating some degree of instability similarly to mouse serum. Barbas et al. reported that DFSA did not bind to single amino acids like Lys, Cys, Ser, Thr, His and Trp.^24^ This highlights the critical role of the tertiary structure of HSA for the binding ability of DFSA. Thus, the varying binding rates of DFSA to mouse vs human albumin can be explained most likely by deviations in their protein structure as already the amino acid sequence is different.^34^ In contrast to **DFSA-Ox-OAc**, in human serum both **PODS-Ox-OAc** and **Mal-Ox-OAc** were stably bound to albumin (∼90%) from 1 to 24 h. However, **Mal-Ox-OAc** exhibited distinctly faster initial binding at the 0 h time point with 29% albumin-bound vs <5% for **PODS-Ox-OAc**. Notably, the lower hydrolytic stability of the different binding units (maleimide < PODS < DFSA; Figure 1) inversely correlates with faster albumin binding in human serum (maleimide > PODS > DFSA; Figure S6 and Table S4). The original publication of Barbas et al. reported a similar binding rate of DFSA compared to maleimide after 2 h incubation for recombinant HSA and in human plasma.^24^ However, the authors used distinctly higher concentrations of DFSA in their incubation studies (50 μM DFSA in 5-fold diluted plasma). Furthermore, when using equimolar DFSA/albumin ratios, they observed less than 25% binding after 24 h and only a 10-fold excess resulted in full albumin binding.^24^

**Figure 2:**
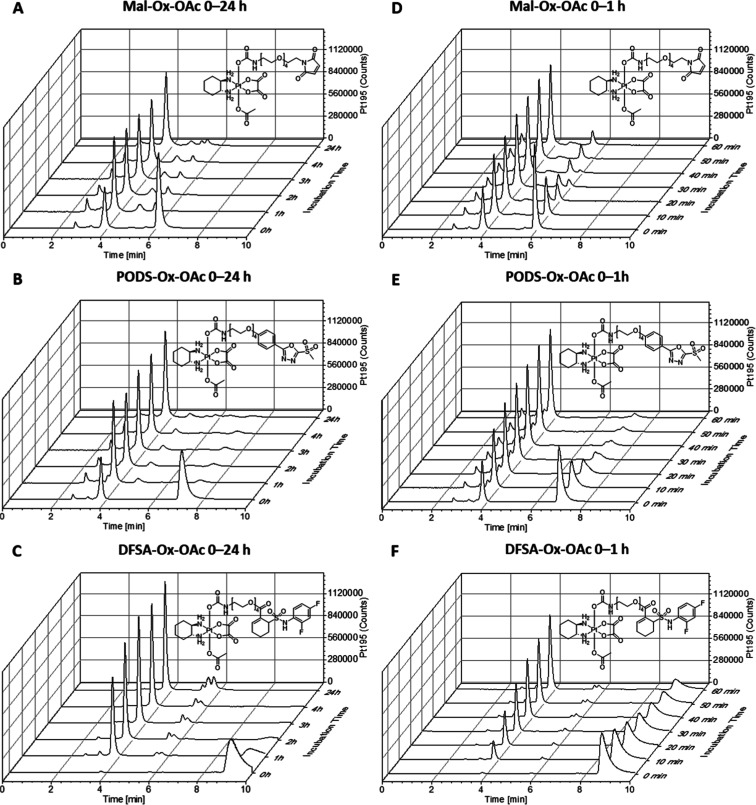
^195^Pt traces of SEC-ICP-MS measurements. The target compounds were incubated at 100 μM in mouse serum buffered with 150 mM phosphate at 37 °C and pH 7.4. The samples containing (A) **Mal-Ox-OAc**, (B) **PODS-Ox-OAc** or (C) **DFSA-Ox-OAc** were measured every hour for 4 h and again after 24 h. In addition, (D) **Mal-Ox-OAc**, (E) **PODS-Ox-OAc** and (F) **DFSA-Ox-OAc** were measured every 10 min for 1 h, using the same conditions.

Finally, to determine the protein binding specificity profile of the target compounds, incubation studies with other serum proteins were performed. Therefore, four of the most abundant human serum proteins (albumin, IgG, α1-antitrypsin and apotransferrin)^35^ were incubated with **Mal-Ox-OAc**, **PODS-Ox-OAc** and **DFSA-Ox-OAc** (200 μM protein, 100 μM compound) in 100 mM PB at pH 7.4 and 37 °C for 2 h and subsequently analyzed via SEC-ICP-MS (Figure S7). In the presence of albumin, 75% and 69% platinum were detected in the HMWF for **Mal-Ox-OAc** and **PODS-Ox-OAc**, respectively, whereas only 26% of **DFSA-Ox-OAc** were albumin-bound after 2 h. For IgG, less than 20% **Mal-Ox-OAc** and **PODS-Ox-OAc** were protein-bound and for apotransferrin less than 10%. Of note, an additional peak in the LMWF was observed after the incubation of **PODS-Ox-OAc** with IgG (Figure S7B). **DFSA-Ox-OAc** showed significantly less binding than the other two compounds with <5% platinum in the HMWF for both proteins. With α1-antitrypsin slightly increased protein-binding was observed for all compounds with ∼30% platinum in the HMWF for **Mal-Ox-OAc** and **PODS-Ox-OAc** and 20% for **DFSA-Ox-OAc**. Although **DFSA-Ox-OAc** showed an increased tendency toward albumin binding compared to other serum proteins, the total platinum content in the HMWF remained relatively low, suggesting inefficient protein-binding properties in general.

Taken together, these data clearly indicate that **Mal-Ox-OAc** and **PODS-Ox-OAc** exhibit similar protein-binding profiles, with albumin showing the highest binding efficiency among the proteins tested. Notably, in serum, albumin is by far the most abundant protein, limiting the availability of **Mal-Ox-OAc** and **PODS-Ox-OAc** for interaction with other proteins.

### Biodistribution and Anticancer Activity In Vivo

Next, we investigated how the differing chemical properties and albumin-binding kinetics (observed in the SEC-ICP-MS spiking experiments) impact the pharmacokinetic profile and anticancer activity of the prodrugs in vivo. For this purpose, the murine colorectal cancer model CT26 was utilized. This allograft system exhibits moderate sensitivity to oxaliplatin,^28^ is known for its favorable response to oxaliplatin-releasing, albumin-binding platinum­(IV) prodrugs, and has already been well-characterized with regard to its albumin uptake.^36^ To get more insight into the pharmacokinetics and organ distribution patterns, CT26 cells were inoculated subcutaneously into the right flank of male Balb/c mice. When tumors with a size of ∼150 mm^3^ had formed (day 10), a single equimolar dose of each of the three drugs was administered intravenously (i.v.) and blood, tumor and organs were harvested according to the study design in Figure 3A. **PODS-Ox-OAc** exhibited the highest levels in serum (AUC_Pt_0‑∞_ 638 mg h/kg; AUC_Pt_0–24_ 461 mg h/kg), followed by **Mal-Ox-OAc** (AUC_Pt_0‑∞_ 496 mg h/kg; AUC_Pt_0–24_ 369 mg h/kg) and **DFSA-Ox-OAc** (AUC_Pt_0‑∞_ 64.5 mg h/kg; AUC_Pt_0–24_ 35.1 mg h/kg; Figure 3B). Notably, **DFSA-Ox-OAc** concentrations showed only minor differences compared to oxaliplatin (AUC_Pt_0‑∞_ 37.5 mg h/kg; AUC_Pt_0–24_ 26.6 mg h/kg), suggesting higher initial excretion due to the relatively slow albumin-binding properties of DFSA. A list of all calculated pharmacokinetic parameters can be found in Table S5. Already at 5 min post injection the platinum levels of **PODS-Ox-OAc** and **Mal-Ox-OAc** were significantly higher than those of oxaliplatin (∼9- and ∼6-fold, respectively; Figure 3B). Overall, these results align well with previous reports indicating that albumin-binding enhances drug retention.^11,12^ A notable difference was observed between the two fast-binding complexes, particularly at early time points of the experiment. In more detail, the serum platinum levels of **PODS-Ox-OAc**-treated animals at 5 and 30 min were about 50% higher than the ones after **Mal-Ox-OAc** treatment (Figure 3B). However, after 5 h the plasma platinum levels in both treatment groups converged to similar values. The observed differences in initial serum platinum levels despite similar albumin binding in the SEC-ICP-MS spiking experiments, might be due to other factors which influence tissue distribution and elimination like molecular charge, size, or lipophilicity of the unbound prodrugs. Also, when looking at the organ distribution of the compounds, a strong impact of the fast albumin-binding of **PODS-Ox-OAc** and **Mal-Ox-OAc** in comparison to **DFSA-Ox-OAc** or oxaliplatin was observed (Figure S8). **Mal-Ox-OAc** and **PODS-Ox-OAc** resulted in ∼4-fold enhanced drug accumulation in tumor tissues at both studied time points compared to the slow albumin-binding compounds (Figure 3C). Moreover, there was a trend toward higher tumoral levels in the **PODS-Ox-OAc** group compared to those receiving **Mal-Ox-OAc**. However, the number of analyzed animals was too small to reach statistical significance. Animals treated with **DFSA-Ox-OAc** or oxaliplatin showed up to 6-fold higher platinum concentrations in liver tissue and elevated levels in the kidney compared to the tumor (Figure S9). Interestingly, **Mal-Ox-OAc-**treated animals had (especially at the 5 h time point) a higher kidney-to-tumor ratio than the ones receiving **PODS-Ox-OAc** (∼1.7-fold vs ∼1-fold).

**Figure 3:**
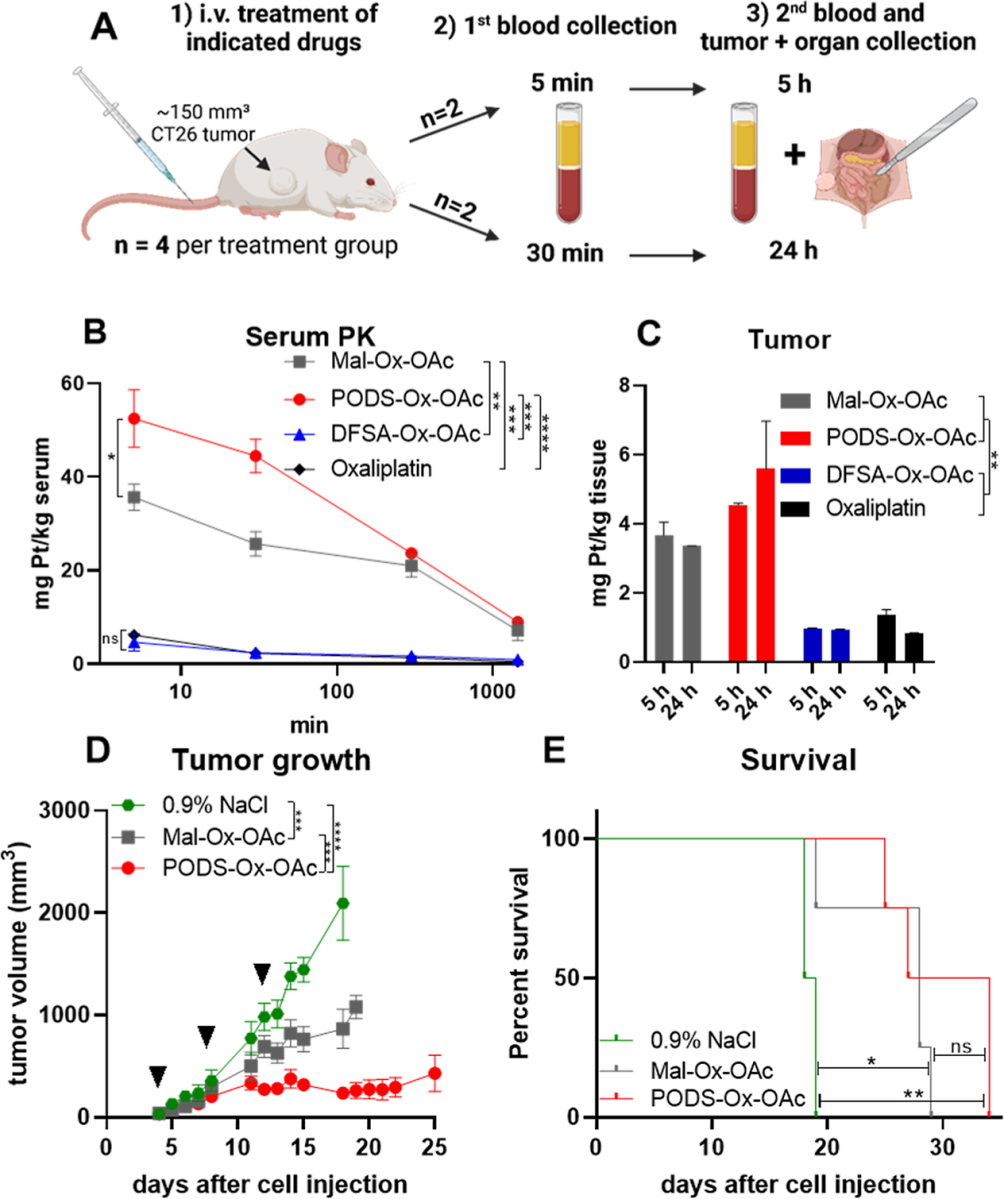
Pharmacological evaluation and anticancer activity of the platinum complexes in CT26-bearing Balb/c mice. For the pharmacological studies, animals were treated once i.v. with doses equimolar to 9 mg/kg oxaliplatin. (A) Scheme of the study design, (B) pharmacokinetic (PK) study: serum samples were collected after 5 min, 30 min, 300 min and 1440 min; (C) tumor samples were collected from 2 mice of each group after 5 and 24 h, respectively. Platinum levels of all samples were measured via ICP-MS. For evaluation of anticancer activity, CT26-bearing Balb/c mice were treated on day 4, 8, and 12 after cell injection (marked with an arrow ▼) i.v. with concentrations equimolar to 9 mg/kg oxaliplatin (= maximum tolerable dose). (D) Impact on tumor growth; data shown until the first animal of the respective treatment group had to be sacrificed. Data given in (B–D) are means ± SEM. Statistical significance was tested by two-way ANOVA (**p < 0.01, ***p < 0.001, ****p < 0.0001, ns = not significant). (E) The overall survival is depicted via a Kaplan–Meier curve. Statistical significance was tested by Log-rank test and Mantel–Cox posttest (*p < 0.05; **p < 0.01; ns = not significant). The oxaliplatin data were used from Schueffl et al.^36^

Finally, the anticancer activity of the two rapid albumin-binders was assessed in CT26-bearing Balb/c mice with overall survival as the primary end point. The rather moderate activity of oxaliplatin in this model, which does not lead to prolonged overall survival, has already been well described.^28,36^ In these studies, **Mal-Ox-OAc** was given four times a week over 2 weeks, which is our standard therapy scheme. However, preliminary tests with the new drugs indicated that **PODS-Ox-OAc** might not have sufficient tolerability in this scheme (data not shown). Consequently, we used a less dense setting in the next experiment, with only three administrations over 2 weeks (days 4, 8, and 12 post cell injection). Similar to other oxaliplatin-releasing maleimide platinum­(IV) drugs,^28,36^ even when given in this reduced treatment regimen, **Mal-Ox-OAc** exhibited strong and highly significant anticancer activity. This was indicated by distinctly reduced tumor growth and significantly prolonged overall survival of about 10 days (∼50% increase) compared to solvent-treated animals (Figure 3D,E). **PODS-Ox-OAc** was well-tolerated in this scheme (Figure S10) and exhibited even superior anticancer efficacy compared to the maleimide-functionalized platinum­(IV) complex (*p* < 0.001). This led to slight remission or stable disease for up to 2 weeks after the last **PODS-Ox-OAc** treatment (Figure S11). The strong anticancer activity also translated into prolonged overall survival compared to the control group (∼80% increase). Thus, **PODS-Ox-OAc**-treated animals exhibited an increased lifespan compared to those that received **Mal-Ox-OAc**. However, due to the small number of tested animals, statistical significance was not reached. Still, these findings indicate that the slightly altered pharmacokinetic profile of **PODS-Ox-OAc** might lead to enhanced tumor targeting and anticancer activity.

## Conclusion

Maleimides are widely used in anticancer drug development for conjugating small-molecule drugs to macromolecules due to their fast and selective reaction with thiols. However, maleimides are unstable under physiological conditions and the thiosuccinimide bond formed during conjugation has limited stability. To overcome these drawbacks, various other conjugating moieties have been developed in the past few years.^17^ However, they are typically just used for ex vivo reactions where nonphysiological conditions and extended incubation times can be applied. This is especially problematic for the development of albumin-binding drugs where in vivo conjugation to endogenous albumin after intravenous administration is required. We herein selected phenyloxadiazolyl methyl sulfone (PODS) and a 2,4-difluorophenyl sulfonamide (DFSA) derivative, which bind to Cys34 and Lys64 of albumin, respectively, for comparison with maleimide. To this end, the three oxaliplatin­(IV) prodrugs **PODS-Ox-OAc**, **DFSA-Ox-OAc** and **Mal-Ox-OAc** were synthesized. **DFSA-Ox-OAc** exhibited the highest hydrolytic stability at pH 7.4, followed by **PODS-Ox-OAc** and **Mal-Ox-OAc**. Albumin-binding studies in mouse and human serum revealed that **DFSA-Ox-OAc** required several hours to achieve maximum binding of 82% and 66%, respectively. In contrast, for **PODS-Ox-OAc,** we observed rapid albumin binding (∼60% after 10 min) in mouse serum and the formation of a stable conjugate over 24 h, well comparable to **Mal-Ox-OAc**. In human serum, **Mal-Ox-OAc** showed distinctly faster binding than **PODS-Ox-OAc** directly after sample preparation. Nevertheless, after 1 h, both compounds were bound to albumin with ∼90%. The strongly divergent results for **DFSA-Ox-OAc** in the different sera can most probably be explained by the importance of the tertiary albumin structure for DFSA-binding, which is essential for the accessibility of Lys64 and varies in different species. Cys34, on the other hand, is known to be situated at the surface of albumin, which substantially facilitates the binding of thiol-conjugating moieties. The hydrolytic stability of the binding moieties (maleimide < PODS < DFSA) was inversely correlated with the binding rate to albumin (maleimide > PODS > DFSA) in human serum. These findings underscore the critical need to balance the reactivity and stability of the binding unit for developing drug-delivery systems that bind to endogenous albumin in vivo.

The serum pharmacokinetic and organ distribution study in CT26-bearing mice showed significantly higher levels of **PODS-Ox-OAc** in serum after 5 and 30 min compared to **Mal-Ox-OAc**. Interestingly, the serum platinum levels of the two fast albumin-binders aligned after 5 h. In contrast, **DFSA-Ox-OAc** exhibited much lower levels, similar to those of oxaliplatin. In good agreement with the reported therapeutic effects of albumin-binding drugs,^11,12^ we observed not only higher general plasma levels but also enhanced tumor-targeting of **PODS-Ox-OAc** and **Mal-Ox-OAc** compared to **DFSA-Ox-OAc** and oxaliplatin. Consequently, the data indicate that **DFSA-Ox-OAc** might bind to albumin too slowly to obtain any beneficial tumor-targeting properties. In vivo studies in CT26-allograft bearing mice revealed a significantly higher inhibition of tumor growth and prolonged overall survival for **PODS-Ox-OAc** compared to **Mal-Ox-OAc**. Overall, this strong difference is surprising, given the minor variations in albumin-binding behavior observed in vitro. One explanation might be that different lipophilicity or charge of the unbound small-molecular platinum­(IV) complex results in differences in their initial serum to tissue equilibrium right after intravenous administration. This could result in a larger hydrolyzed fraction of the maleimide-functionalized drug **Mal-Ox-OAc** compared to **PODS-Ox-OAc** in the tissue, preventing subsequent albumin-binding and, thus, leading to renal excretion of this fraction. However, which parameters exactly influence this process is speculative and needs to be addressed in more detailed pharmacological studies in the future.

Summarizing, in this study we show the potential of the binding unit PODS as a promising candidate and viable alternative to maleimide for the development of anticancer drug delivery systems that exploit covalent binding to endogenous albumin in vivo.

## Methods

### Chemicals

(*OC*-6–44)-Acetato­[(1*R*,2*R*)-1,2-cyclohexane-1,2-diamino]­oxalato hydroxyplatinum­(IV) (OxOAcOH),^29^ 4-(5-(methylsulfonyl)-1,3,4-oxadiazol-2-yl)­phenol (PODS),^30^ 6-(*N*-(2,4-difluorophenyl)­sulfamoyl)­cyclohex-1-ene-1-carboxylic acid (DFSA),^24,31^ and 1-(14-isocyanato-3,6,9,12-tetraoxatetradecyl)-1*H*-pyrrole-2,5-dione (Mal-PEG_4_-NCO)^33^ were synthesized according to literature procedures. Milli-Q water (18.2 MΩ cm, Merck Milli-Q Advantage, Darmstadt, Germany) was used for synthesis and for analytical and preparative RP-HPLC. Potassium tetrachloridoplatinate (K_2_[PtCl_4_]) was purchased from Johnson Matthey (Switzerland) and anhydrous solvents (DMF, EtOAc, THF and toluene) over molecular sieves from Acros Organics. All other chemicals and solvents were purchased from commercial suppliers (abcr, Alfa Aesar, Ambeed, BLDpharm, Acros Organics/Fisher Scientific, EGA-Chemie, Sigma-Aldrich, Th. Geyer, Toronto Research Chemicals) and used without purification. Silica gel (particle size 40–63 μm) for column chromatography was purchased from VWR. High-resolution mass spectra were measured on a Bruker maXis UHR ESI time-of-flight mass spectrometer in positive mode by direct infusion at the Mass Spectrometry Centre of the University of Vienna. ^1^H NMR spectra were recorded at 25 °C using a Bruker FT-NMR spectrometer AV NEO 500 at 500.10 MHz. Only for the characterization of final compounds **Mal-Ox-OAc**, **PODS-Ox-OAc** and **DFSA-Ox-OAc** one- and two-dimensional NMR spectra were recorded at 25 °C using a Bruker FT-NMR AVIII 600 MHz spectrometer. ^1^H NMR spectra were measured at 600.25 MHz and ^13^C NMR spectra at 150.93 MHz. All NMR spectra were measured in deuterated dimethyl sulfoxide (DMSO-*d*_6_). Chemical shifts (ppm) were referenced internally to the residual solvent peaks. For the description of the spin multiplicities the following abbreviations were used: s = singlet, d = doublet, t = triplet, q = quartet, bs = broad singlet, dd = doublet of doublets, ddd = doublet of doublet of doublets, td = triplet of doublets, m = multiplet. The ^1^H and ^13^C NMR spectra of the final compounds are depicted in Figures S12–S17. Analytical HPLC runs of all final compounds were performed on the same instrument as stability and reduction measurements and are depicted in Figure S18. Purification by preparative reverse-phase (RP) HPLC was performed on an XBridge BEH C18 OBD Prep Column (19 mm × 250 mm) on an Agilent 1260 Infinity II system. Flow rates of 17 mL/min were constant for each run at 20 °C. Elemental analysis measurements were performed on a Eurovector EA 3000 CHNS-O Elemental Analyzer at the Microanalytical Laboratory of the University of Vienna and are within ±0.4%, confirming >95% purity.

### Synthesis

*Tert*-butyl (2-(2-(2-(2-(4-(5-(methylsulfonyl)-1,3,4-oxadiazol-2-yl)­phenoxy)­ethoxy)­ethoxy)­ethoxy)­ethyl)­carbamate (PODS-PEG_4_-NHBoc): Ph_3_P on polymer (1.93 g, 3.09 mmol, 1.5 equiv; 1.6 mmol/g loading on styrol cross-linked with 1% divinylbenzene) was suspended in a mixture of 11.5 mL dry THF and 11.5 mL dry toluene and DIAD (621 μL, 3.09 mmol, 1.5 equiv) was added at room temperature. The mixture was cooled below 0 °C with NaCl/ice and a suspension containing PODS (500 mg, 2.06 mmol, 1.0 equiv), HO-PEG_4_-NHBoc (935 mg, 3.09 mmol, 1.5 equiv) and neopentyl alcohol (92 mg, 1.03 mmol, 0.5 equiv) in a mixture of 5.3 mL dry THF, 5.3 mL dry toluene and 0.8 mL dry DMF was added over 30 min. The mixture was warmed to room temperature and stirred for 72 h. The polymer was filtered off, washed with THF and DCM and the filtrate was concentrated to yield 1.98 g of a light-orange oil. The crude was purified via silica column chromatography with EtOAc/Hex using an 80–85% EtOAc gradient. PODS-PEG_4_-NHBoc was obtained as a waxy white solid in 50% yield (535 mg, 1.04 mmol). ^1^H NMR (500 MHz, DMSO): δ 8.05–8.01 (m, 2H), 7.23–7.19 (m, 2H), 6.77 (t, *J* = 5.5 Hz, 1H), 4.25–4.20 (m, 2H), 3.80–3.77 (m, 2H), 3.70 (s, 3H), 3.62–3.58 (m, 2H), 3.56–3.53 (m, 2H), 3.53–3.47 (m, 4H), 3.39–3.35 (m, 2H, overlap with H_2_O), 3.05 (q, *J* = 6.0 Hz, 2H), 1.36 (s, 9H) ppm.

2-(2-(2-(2-(4-(5-(Methylsulfonyl)-1,3,4-oxadiazol-2-yl)­phenoxy)­ethoxy)­ethoxy)­ethoxy)­ethan-1-aminium trifluoroacetate (PODS-PEG_4_-NH_2_): PODS-PEG_4_-NHBoc (533 mg, 1.03 mmol, 1.0 equiv) was dissolved in 16 mL dry DCM and TFA (2.8 mL, 36.2 mmol, 35 equiv) was added at room temperature The mixture was stirred for 35 min, then all volatiles were removed. The oily residue was suspended in H_2_O and lyophilized to give PODS-PEG_4_-NH_2_ as a white solid in 97% yield (531 mg, 1.03 mmol). ^1^H NMR (500 MHz, DMSO): δ 8.04 (d, *J* = 8.9 Hz, 2H), 7.75 (br s, 3H), 7.21 (d, *J* = 8.9 Hz, 2H), 4.25–4.20 (m, 2H), 3.81–3.76 (m, 2H), 3.70 (s, 3H), 3.62–3.55 (m, 10H), 2.97 (t, *J* = 5.2 Hz, 2H) ppm.

1-(6-(*N*-(2,4-Difluorophenyl)­sulfamoyl)­cyclohex-1-en-1-yl)-1-oxo-2,5,8,11-tetraoxatridecan-13-aminium trifluoroacetate (DFSA-PEG_4_-NH_2_): Ph_3_P on polymer (281 mg, 0.45 mmol, 1.5 eq.; 1.6 mmol/g loading on styrol cross-linked with 1% divinylbenzene) was suspended in a mixture of 1.7 mL dry THF and 1.7 mL dry toluene and DIAD (90 μL, 0.45 mmol, 1.5 equiv) was added at room temperature. The mixture was cooled below 0 °C with NaCl/ice and a solution containing DFSA (95 mg, 0.30 mmol, 1.0 equiv), HO-PEG_4_-NHBoc (136 mg, 0.45 mmol, 1.5 equiv) and neopentyl alcohol (13 mg, 0.15 mmol, 0.5 equiv) in a mixture of 0.8 mL dry THF and 0.8 mL dry toluene was added over 15 min. The mixture warmed to room temperature and stirred for 68 h. The polymer was filtered off, washed with THF and the filtrate was concentrated to yield 354 mg of a light-orange oil. The crude was dissolved in 4.5 mL DCM and TFA (0.8 mL, 10.36 mmol) was added at room temperature. The mixture was stirred for 40 min, then all volatiles were removed. The oily residue was purified via isocratic preparative RP-HPLC (C18 X-bridge) using MeCN/Milli-Q-H_2_O 30:70 with 0.1% TFA as the eluent. DFSA-PEG_4_-NH_2_ was obtained as a clear oil in 57% yield (104 mg, 0.17 mmol). ^1^H NMR (500 MHz, DMSO): δ 9.85 (s, 1H), 7.73 (br s, 2H), 7.48 (td, *J* = 9.1, 6.1 Hz, 1H), 7.36 (ddd, *J* = 10.9, 9.2, 2.8 Hz, 1H), 7.14–7.07 (m, 2H), 4.28 (d, *J* = 5.1 Hz, 1H), 4.16–4.03 (m, 2H), 3.59–3.48 (m, 12H), 3.02–2.91 (m, 2H), 2.42–2.32 (m, 3H), 2.26–2.17 (m, 1H), 2.12–2.01 (m, 1H), 1.78–1.69 (m, 1H), 1.66–1.58 (m, 1H) ppm.

(*OC*-6–34)-Acetato­[(1*R*,2*R*)-1,2-cyclohexane-1,2-diamino]­oxalato­(2,5-dioxopyrrolidin-1-yl carbonato) platinum­(IV) (OxOAcNHS): OxOAcOH (200 mg, 0.42 mmol, 1.0 equiv) was suspended in 18 mL DMF and *N*,*N*′-disuccinimidyl carbonate (DSC; 166 mg, 0.63 mmol, 1.5 equiv) was added. The mixture was stirred at room temperature for 24 h, then the solvent was removed. The residue was taken up in 10 mL MeCN and the product precipitated with Et_2_O. The solid was filtered and washed 2× with Et_2_O. The filtrate was concentrated and a second and third fraction were collected likewise. OxOAcNHS was obtained as an off-white solid in 90% yield (234 mg, 0.38 mmol). ^1^H NMR (500 MHz, DMSO): δ 8.55 (d, *J* = 7.7 Hz, 1H), 8.39 (d, *J* = 7.5 Hz, 1H), 8.16 (t, *J* = 8.9 Hz, 1H), 7.93 (d, *J* = 9.1 Hz, 1H), 2.73 (s, 4H), 2.57–2.53 (m, 2H, overlap with DMSO), 2.14–2.04 (m, 2H), 1.96 (s, 3H), 1.60–1.39 (m, 4H), 1.20–1.10 (m, 2H) ppm.

(*OC*-6–34)-Acetato­[(1*R*,2*R*)-1,2-cyclohexane-1,2-diamino]­[(2-(2-(2-(2-(4-(5-(methylsulfonyl)-1,3,4-oxadiazol-2-yl)­phenoxy)­ethoxy)­ethoxy)­ethoxy)­ethyl)­carbamato]­oxalato platinum­(IV) (**PODS-Ox-OAc**): OxOAcNHS (55 mg, 0.085 mmol, 1.0 equiv) was dissolved in 2.7 mL dry DMF and a solution of PODS-PEG_4_-NH_2_ (61 mg, 0.12 mmol, 1.4 equiv) and NEt_3_ (60 μL, 0.42 mmol, 5 equiv) in 2.7 mL dry DMF was added. The mixture was stirred for 1.5 h and all volatiles were removed. The residue was purified via isocratic preparative RP-HPLC (C18 X-bridge) using MeCN/Milli-Q-H_2_O 28:72 with 0.1% TFA as the eluent. PODS-Ox-OAc was obtained as a white solid in 41% yield (35 mg, 0.035 mmol). ^1^H NMR (600 MHz, DMSO): δ 9.82–9.30 (m, 1H, H4), 8.57 (d, *J* = 5.6 Hz, 1H, H4), 8.27 (br s, 2H, H4), 8.04 (d, *J* = 8.7 Hz, 2H, H20), 7.21 (d, *J* = 8.7 Hz, 2H, H19), 6.70 (t, *J* = 5.00 Hz, 1H, H9), 4.25–4.20 (m, 2H, H17), 3.80–3.76 (m, 2H, H16), 3.70 (s, 3H, H24), 3.62–3.52 (m, 8H, overlap with H_2_O, H12–15), 3.35 (t, *J* = 5.2 Hz, 2H, H11), 3.11–2.99 (m, 2H, H10), 2.56 (br s, 2H, H5), 2.12 (t, *J* = 9.3 Hz, 2H, H6), 1.95 (s, 3H, H1), 1.50 (d, *J* = 10.0 Hz, 2H, H7), 1.46–1.31 (m, 2H, H6), 1.21–1.08 (m, 2H, H7) ppm. ^13^C NMR (151 MHz, DMSO): δ 178.35 (C2), 165.75 (C22), 164.19 (C8), 163.37 + 163.28 (2C, C3), 162.28 (C18), 161.67 (C23), 129.41 (C20), 115.57 (C19), 114.25 (C21), 69.91 + 69.76 + 69.48 (4C, C12–15), 69.21 (C11), 68.69 (C16), 67.68 (C17), 61.15 + 60.80 (2C, C5), 42.90 (C24), 40.65 (C10), 30.95 + 30.81 (2C, C6), 23.56 + 23.44 (2C, C7), 22.88 (C1) ppm. HRMS (*m*/*z*): calcd. C_28_H_41_N_5_O_15_PtS (M + Na)^+^, 937.1862; found, 937.1851. Elemental analysis (%): calcd. for C_28_H_41_N_5_O_15_PtS*H_2_O*0.5TFA, C: 35.19, H: 4.43, N: 7.08, S: 3.24; found, C: 34.93, H: 4.32, N: 7.09, S: 3.03.

(*OC*-6–34)-Acetato­[(1*R*,2*R*)-1,2-cyclohexane-1,2-diamino]­[(1-(6-(*N*-(2,4-difluorophenyl)­sulfamoyl)­cyclohex-1-en-1-yl)-1-oxo-2,5,8,11-tetraoxatridecan-13-yl)­carbamato]­oxalato platinum­(IV) (**DFSA-Ox-OAc**): DFSA-PEG_4_-NH_2_ (42 mg, 0.070 mmol, 1.0 equiv) was dissolved in 2.0 mL dry DMF and OxOAcNHS (43 mg, 0.070 mmol, 1.0 equiv) and NEt_3_ (97 μL, 0.69 mmol, 10 equiv) were added. The mixture was stirred for 20 h, after which additional OxOAcNHS (9 mg, 0.15 mmol, 0.2 equiv) was added. The mixture was stirred for another 4 h at room temperature, then all volatiles were removed. The residue was purified via isocratic preparative RP-HPLC (C18 X-bridge) using MeCN/Milli-Q-H_2_O 35:65 with 0.1% TFA as the eluent. DFSA-Ox-OAc was obtained as a white solid in 19% yield (14 mg, 0.013 mmol). ^1^H NMR (600 MHz, DMSO): δ 9.83 (s, 1H, H25), 9.79–9.37 (m, 1H, H4), 8.58 (d, *J* = 6.2 Hz, 1H, H4), 8.27 (br s, 2H, H4), 7.48 (td, *J* = 9.1, 6.1 Hz, 1H, H27), 7.35 (ddd, *J* = 11.6, 9.0, 2.9 Hz, 1H, H30), 7.14–7.12 (m, 1H, H20), 7.12–7.08 (m, 1H, H28), 6.71 (t, *J* = 5.2 Hz, 1H, H9), 4.28 (d, *J* = 5.3 Hz, 1H, H24), 4.14–4.03 (m, 2H, H17), 3.57–3.52 (m, 2H, H16), 3.51–3.46 (m, 8H, overlap with H_2_O, H12–15), 3.34 (t, *J* = 5.9 Hz, 2H, H11), 3.11–2.99 (m, 2H, H10), 2.56 (br s, 2H, H5), 2.41–2.31 (m, 2H, H21, H23), 2.26–2.18 (m, 1H, H21), 2.12 (t, *J* = 12.4 Hz, 2H, H6), 2.10–2.03 (m, 1H, H22), 1.95 (s, 3H, H1), 1.78–1.69 (m, 1H, H23), 1.65–1.58 (m, 1H, H22), 1.50 (d, *J* = 10.2 Hz, 2H, H7), 1.46–1.32 (m, 2H, H6), 1.21–1.08 (m, 2H, H7) ppm. ^13^C NMR (151 MHz, DMSO): δ 178.39 (s, C2), 165.65 (s, C18), 164.21 (s, C8), 163.39 + 163.32 (s, 2C, C3), 159.53 (dd, *J* = 245.0, 11.3 Hz, C31), 155.54 (dd, *J* = 249.1, 12.7 Hz, C29), 145.88 (s, C20), 127.44 (d, *J* = 9.9 Hz, C27), 123.77 (s, C19), 121.62 (dd, *J* = 13.0, 3.6 Hz, C26), 111.64 (dd, *J* = 22.2, 3.6 Hz, C28), 104.60 (dd, *J* = 26.7, 24.4 Hz, C30), 69.74 + 69.72 + 69.64 + 69.47 (s, 4C, C12–15) 69.23 (s, C11), 68.08 (s, C16), 63.66 (s, C17), 61.16 + 60.81 (s, 2C, C5), 54.97 (s, C24), 40.63 (s, C10), 30.96 + 30.82 (s, 2C, C6), 24.42 (s, C21), 23.58 + 23.46 (s, 2C, C7), 22.91 (s, C1), 22.17 (s, C23), 15.90 (s, C22) ppm. HRMS (*m*/*z*): calcd. C_32_H_46_F_2_N_4_O_15_PtS (M + Na)^+^, 1014.2191; found, 1014.2165. Elemental analysis (%): calcd. for C_32_H_46_F_2_N_4_O_15_PtS*H_2_O*0.5TFA, C: 37.15, H: 4.58, N: 5.25, S: 3.00; found, C: 37.18, H: 4.44, N: 5.35, S: 2.94.

(*OC*-6–34)-Acetato­[(1*R*,2*R*)-1,2-cyclohexane-1,2-diamino]­[ (14-(2,5-dioxo-2,5-dihydro-1*H*-pyrrol-1-yl)-3,6,9,12-tetraoxatetradecyl)­carbamato]­oxalato platinum­(IV) (**Mal-Ox-OAc**): OxOAcOH (140 mg, 0.30 mmol, 1.0 equiv) and Mal-PEG_4_-NCO (160 mg, 0.45 mmol, 1.5 equiv) were dissolved in 3 mL dry DMF and stirred overnight at room temperature. The solvent was removed and the crude product was purified via isocratic preparative RP-HPLC (C18 X-bridge) using MeCN/Milli-Q-H_2_O 14:86 as the eluent. Mal-Ox-OAc was obtained as an orange solid in 22% yield (55 mg, 0.066 mmol). ^1^H NMR (600 MHz, DMSO): δ 9.78–9.35 (m, 1H, H4), 8.57 (d, *J* = 6.7 Hz, 1H, H4), 8.27 (br s, 2H, H4), 7.02 (s, 2H, H21), 6.71 (t, *J* = 5.4 Hz, 1H, H9), 3.57–3.54 (m, 2H, H19), 3.52–3.50 (m, 2H, H18), 3.50–3.43 (m, 14H, H11–17), 3.11–3.00 (m, 2H, H10), 2.60–2.53 (br s, 2H, H5), 2.13 (t, *J* = 9.7 Hz, 2H, H6), 1.95 (s, 3H, H1), 1.51 (d, *J* = 10.2 Hz, 2H, H7), 1.47–1.30 (m, 2H, H6), 1.21–1.09 (m, 2H, H7) ppm. ^13^C NMR (151 MHz, DMSO): δ 178.42 (C2), 170.94 (2C, C20), 164.21 (C8), 163.42 + 163.36 (2C, C3), 134.58 (2C, C21), 69.79 + 69.74 + 69.64 + 69.51 + 69.41 + 69.25 (7C, C11–17), 66.94 (C18), 61.18 + 60.83 (2C, C5), 40.66 (C10), 36.82 (C19), 30.98 (C6), 30.85 (C6), 23.60 (C7), 23.48 (C7), 22.93 (C1) ppm. HRMS (*m*/*z*): calcd. C_25_H_40_N_4_O_14_Pt (M + H)^+^, 816.2264; found, 816.2266. Elemental analysis (%): calcd. for C_25_H_40_N_4_O_14_Pt*H_2_O, C: 36.02, H: 5.08, N: 6.72; found, C: 36.08, H: 4.94, N: 6.71.

### Biology/Analytics

#### UHPLC/LCMS Stability Measurements

**PODS-Ox-OAc**, **DFSA-Ox-OAc** and **Mal-Ox-OAc** were dissolved at 0.5 mM in 100 mM PB at pH 7.4. The samples were incubated at 20 °C and measured over 25 h. Reversed phase high-performance liquid chromatography (RP-HPLC) runs were performed using a Waters Acquity UPLC BEH C18 column (130 Å, 1.7 μm, 3 mm × 50 mm) on a Dionex Thermo Scientific UltiMate 3000 HPLC system, equipped with a HPG-3400RS binary pump and a DAD-3000 UV–vis detector. Chromatograms were evaluated at a wavelength of 220 nm. Milli-Q water (mobile phase A) and acetonitrile (mobile phase B), both containing 0.1% TFA, were used as eluents. The flow rate was consistent at 0.6 mL/min for all measurements using the following gradient: 0–0.5 min A 95:5 B, 0.5–5.5 min linear gradient to A 5:95 B, 5.5–6.5 min A 5:95 B, 6.5–9.2 min A 95:5 B. The same conditions were applied for stability measurements in 100 mM PB at pH 5.5 and 6.5 as well as in 20 mM HEPES and MOPS at pH 7.4. RP-HPLC-MS analyses of the same samples after the stability measurements were conducted on a Waters Acquity UPLC BEH C18 column (130 Å, 1.7 μm, 3 mm × 50 mm) on an Agilent 1260 Infinity II system equipped with a Flexible pump, a 1260 VWD UV–vis detector and the LC–MSD system. Chromatograms were evaluated at a wavelength of 220 nm. Milli-Q water (mobile phase A) and acetonitrile (mobile phase B), both containing 0.1% formic acid, were used as eluents. The flow rate was consistent at 0.6 mL/min for all measurements using the following gradient: 0–0.5 min A 95:5 B, 0.5–6.0 min linear gradient to A 5:95 B, 6.0–7.0 min A 5:95 B, 7.0–7.1 min linear gradient to A 95:5 B, 7.1–8.0 min A 95:5 B.

#### UHPLC Reduction Measurements

**PODS-Ox-OAc**, **DFSA-Ox-OAc** and **Mal-Ox-OAc** were dissolved at 0.5 mM in 150 mM PB at pH 7.4 with 30 equiv of ascorbic acid. The samples were incubated at 20 °C and measured over 25 h. To quantify oxaliplatin, a 10 mM stock solution in Milli-Q water was prepared from which a calibration curve of 7 data points between 5 and 500 μM was generated. RP-HPLC runs were performed using a Waters Acquity UPLC HSS T3 column (100 Å, 1.8 μm, 3.0 mm × 50 mm) on a Dionex Thermo Scientific UltiMate 3000 HPLC system, equipped with a HPG-3400RS binary pump and a DAD-3000RS UV–vis detector. Chromatograms were evaluated at a wavelength of 230 nm. Milli-Q water (mobile phase A) and acetonitrile (mobile phase B), both containing 0.1% TFA, were used as eluents. The flow rate was consistent at 0.6 mL/min for all measurements using the following gradient: 0–0.5 min A 99:1 B, 0.5–5.5 min linear gradient to A 1:99 B, 5.5–6.5 min A 1:99 B, 6.5–9.2 min A 99:1 B.

#### SEC-ICP-MS Studies in Serum

Blood of Bab/c mice was collected under anesthesia via heart puncture. The serum was separated by centrifugation (two times 10 min at 900 g) after 10–20 min clotting at room temperature. Human serum was purchased from Sigma-Aldrich. In order to guarantee a stable pH, all sera were buffered with 150 mM phosphate to pH 7.4 by dissolving the necessary amounts of Na_2_HPO_4_ and NaH_2_PO_4_ directly in the serum. **PODS-Ox-OAc**, **DFSA-Ox-OAc** and **Mal-Ox-OAc** were dissolved at 1 mM in 100 mM PB at pH 7.4 and diluted 1:10 in the buffered serum to obtain a final concentration of 100 μM. The samples were then incubated in the autosampler at 37 °C and analyzed every hour for 4 or 5 h and after 24 h. Between each sample a pure water blank was measured. For measurements with 10 min intervals the same procedure was followed, but the blank measurements were omitted. For SEC-ICP-MS measurements an Agilent 1260 Infinity system coupled to an Agilent 7800 ICP-HRMS equipped with a dynamic reaction cell was used. Oxygen (purity 5.5, Messer Austria GmbH, Gumpoldskirchen, Austria) was used as reaction gas. HPLC and column parameters are given in Table S6 and ICP-MS operation parameters are given in Table S7.

### SEC-ICP-MS Studies with Single Serum Protein Solutions

All investigated proteins were purchased from commercial suppliers: Albumin (Sigma-Aldrich, A8763), IgG (Athens Research, 16–16–090707), α1-antitrypsin (Sigma-Aldrich, A6150) and apotransferrin (Sigma-Aldrich, T1147). 200 μM solutions of the proteins in 100 mM PB (pH 7.4) and 1 mM solutions of **Mal-Ox-OAc**, **PODS-Ox-OAc** and **DFSA-Ox-OAc** in Milli-Q water were prepared. The compound stocks were diluted into the protein solution to reach a final concentration of 100 μM (27 μL protein and 3 μL compound). The samples were incubated at 37 °C for 2 h and analyzed as described for the SEC-ICP-MS studies (Tables S6 and S7).

### Cell Culture

For in vivo experiments the murine (Balb/c) CT26 colon cancer cells were used (obtained from American Type Culture Collection), which were cultured in DMEM/F-12 (1:1) medium under standard tissue culture conditions. The media were supplemented with 10% FCS (purchased from PAA Linz, Austria). The cells were not treated with antibiotics and were regularly checked for mycoplasma contamination. All cell culture media and reagents were purchased from Sigma-Aldrich Austria.

#### Animals

Eight- to 12-week-old Balb/c mice were purchased from Envigo Laboratories (San Pietro al Natisone, Italy). The animals were kept in a pathogen-free environment with 12 h light–dark cycle and every procedure was done in a laminar airflow cabinet. All experiments were approved by the Ethics Committee for the Care and Use of Laboratory Animals at the Medical University Vienna (BMWF-2022–0.770.291) and performed according to the guidelines from the Austrian Animal Science Association and from the Federation of European Laboratory Animal Science Associations (FELASA).

#### Pharmacokinetic and Tissue Distribution Studies in CT26-Bearing Balb/C Mice

CT26 cells (5 × 10^5^ in 50 μL serum-free medium) were injected subcutaneously (s.c.) into the right flank of male Balb/c mice. On day 10 after cell injection, when measurable tumors of about 150 mm^3^ had formed, the drugs were administered i.v. (*n* = 4 per treatment group) at a single dose equimolar to 9 mg/kg oxaliplatin dissolved in 0.9% NaCl. The blood, tumor and organ collection followed the study design in Figure 2A. Briefly, to assess serum platinum levels, blood was collected after 5 min, 30 min, 5 h and 24 h (2 mice per time point) via the facial vein. The serum was separated by centrifugation (two times 10 min at 900 g) after 10 min clotting at room temperature. The platinum levels in tumor and organs (liver, kidney, lung, spleen, brain) were determined after 5 h and 24 h (2 animals per time point). To this end, the animals were sacrificed by cervical dislocation, followed by harvesting of the tissues. All collected samples were stored at −20 °C and further processed for platinum measurements via ICP-MS as follows: HNO_3_ (67–69%, suprapur for trace metal analysis, NORMATOM; Distributor: VWR international, Austria) and conc. H_2_O_2_ suprapur (Merck, 30%) were used without further purification. Digestion of tissue (approximately 15–30 mg gravimetrically weighted) was performed with 2 mL of approximately 20% HNO_3_ and 100 μL H_2_O_2_ using an open vessel graphite digestion system (coated graphite heating plate, coated sample holder-top for 25 mL vials, PFA vials and PFA lids; Labter, ODLAB; Distributor: AHF Analysentechnik AG; Germany). Digested samples were diluted in Milli-Q water (18.2 MΩ cm, Milli-Q Advantage, Darmstadt, Germany). For the ICP-MS analysis, platinum and rhenium standards were derived from CPI International (Amsterdam, The Netherlands). The total platinum content was determined with a quadrupole-based Agilent 7800 ICP-MS instrument (Agilent Technologies, Tokyo, Japan) equipped with the Agilent SPS 4 autosampler (Agilent Technologies, Tokyo, Japan) and a MicroMist nebulizer at a sample uptake rate of approximately 0.2 mL/min. An RF power of 1550 W was used as well as nickel cones. Argon was used as plasma gas (15 L/min) and as carrier gas (∼1.1 L/min). The dwell time was set to 0.1 s and the measurements were performed in 6 replicates with 100 sweeps. Rhenium served as internal standard for platinum. The Agilent MassHunter software package (Workstation Software, version C.01.04) was used for data processing.

#### Pharmacokinetic calculations

Noncompartmental pharmacokinetic analysis was performed for median pharmacokinetic profiles in serum (based on 2–4 samples per time point) of **Mal-Ox-OAc**, **PODS-Ox-OAc**, **DFSA-Ox-OAc** and oxaliplatin^36^ using Phoenix WinNonlin 8.5.2.4 (Certara USA, Inc.). As platinum was measured in serum, equimolar administered doses were adjusted to correspond to platinum (0.0227 mmol, 4.42 mg platinum). The area under the “concentration”–time curve (AUC) was determined from time 0 to infinity (AUC_Pt_0‑∞_) and from time 0 to 24 h (AUC_Pt_0–24_, based on the last observed sample at 24 h; linear-log trapezoidal calculation method). The terminal slope (λ_*z*_) was determined based on the last two (out of four) measurements (times 5 h and 24 h).

#### Anticancer Activity Experiment

CT26 cells (5 × 10^5^ in serum-free medium) were injected s.c. into the right flank of male Balb/c mice. Therapy of **Mal-Ox-OAc** and **PODS-Ox-OAc** started when tumor nodules were palpable (day 4). Animals were treated i.v. with the indicated drugs (dissolved in 0.9% NaCl) at a dose equimolar to 9 mg/kg oxaliplatin on day 4, 8, and 12 after cell injection. 0.9% NaCl served as solvent control. Animals were controlled for distress development every day and tumor size was assessed regularly by caliper measurement. Tumor volume was calculated using the formula: (length × width^2^)/2. In the overall survival experiments, mice were sacrificed by cervical dislocation in the case of a tumor length >20 mm, tumor ulceration or body weight loss >20%.

## Supplementary Material



## Notes

HPLC-chromatograms, corresponding mass spectra and tabulated results of stability and reduction measurements; details on SEC-ICP-MS measurements in serum and single protein solutions; pharmacokinetic parameters, details on in vivo organ distribution and activity experiments; NMR spectra and structures of all target compounds (PDF)
