# Supplementary material for: Comparative Evaluation of Thiol- and Amine-Conjugating Moieties for Endogenous Albumin Binding after Intravenous Administration
Source: ACS Pharmacol Transl Sci. 2025 Jun 26;8(7):2192–203. doi: 10.1021/acsptsci.5c00240 (PMC12261219; doi:10.1021/acsptsci.5c00240)
Supplement: Supplementary file 1 [file pt5c00240_si_001.pdf]

# Supporting Information

# Comparative Evaluation of Thiol- and Amine-Conjugating Moieties for Endogenous Albumin Binding after Intravenous Administration

Anja Federa[a,b], Hemma Schueffl[c], Iris K. Minichmayr[d], Alexander Kastner[a,b], Julia Kronberger[a,b,e], Thomas L. Mindt[a,e], Petra Heffeter[c,f] and Christian R. Kowol[a,e, f*]

[a] Institute of Inorganic Chemistry, Faculty of Chemistry, University of Vienna, Waehringer Str. 42, A-1090 Vienna, Austria.
[b] Vienna Doctoral School in Chemistry, University of Vienna, Waehringer Str. 42, 1090 Vienna, Austria.
[c] Center for Cancer Research and Comprehensive Cancer Center, Medical University of Vienna, Borschkegasse 8a, A-1090 Vienna, Austria
[d] Department of Clinical Pharmacology, Medical University of Vienna, Waehringer Guertel 18-20, A-1090 Vienna, Austria
[e] Joint Applied Medicinal Radiochemistry Facility of the University of Vienna and the Medical University Vienna, Vienna, Austria
[f] Research Cluster "Translational Cancer Therapy Research", 1090 Vienna, Austria
Corresponding author: E-Mail: christian.kowol@univie.ac.at

## Table of Contents


| | | |
|---|---|---|
| Figure S1 | HPLC-MS chromatograms after the stability measurements in Figure 1. | Page S-3 |
| Figure S2 | Stability measurements in different pH and buffer conditions. | Page S-5/S-6 |
| Table S1 | Tabulated results of the stability measurements in Figure S2. | Page S-7 |
| Figure S3 | HPLC-MS chromatogram of **PODS-Ox-OAc** after the stability measurement in 20 mM HEPES in Figure S2C. | Page S-7 |
| Figure S4 | Reduction behavior at pH 7.4 in presence of ascorbic acid. | Page S-8 |
| Table S2 | Stability of Oxaliplatin at pH 7.4 over 24 h. | Page S-9 |
| Table S3 | Percentages of $Pt^{195}$ detected in the HMWF and LMWF of SEC-ICP-MS measurements in mouse serum. | Page S-9 |

| | | |
|---|---|---|
| Figure S5 | $Pt^{195}$ trace of the **Mal-Ox-OAc** 10 min timepoint from the SEC-ICP-MS measurement in Figure 2D. | Page S-10 |
| Figure S6 | SEC-ICP-MS measurements in human serum over 24 h. | Page S-11 |
| Table S4 | Percentages of $Pt^{195}$ detected in the HMWF and LMWF of SEC-ICP-MS measurements in human serum. | Page S-11 |
| Figure S7 | Incubation studies with different human serum proteins. | Page S-12/S-13 |
| Table S5 | Pharmacokinetic parameters based on serum platinum levels in mice. | Page S-14 |
| Figure S8 | Organ distribution (liver, kidney, lung, spleen and brain) of the platinum complexes in CT-26-bearing Balb/c mice. | Page S-14 |
| Figure S9 | Ratio of organ to tumor distribution of the platinum complexes in CT-26-bearing Balb/c mice. | Page S-15 |
| Figure S10 | Changes in body weight during therapy. | Page S-16 |
| Figure S11 | Tumor volume of the individual CT26-bearing mice after i.v. treatment of the indicated drugs. | Page S-16 |
| Figure S12 | Structure and $^{1}H$ NMR spectrum of **PODS-Ox-OAc**. | Page S-17 |
| Figure S13 | $^{13}C$ NMR spectrum of **PODS-Ox-OAc**. | Page S-17 |
| Figure S14 | Structure and $^{1}H$ NMR spectrum of **DFSA-Ox-OAc**. | Page S-18 |
| Figure S15 | $^{13}C$ NMR spectrum of **DFSA-Ox-OAc**. | Page S-18 |
| Figure S16 | Structure and $^{1}H$ NMR spectrum of **Mal-Ox-OAc**. | Page S-19 |
| Figure S17 | $^{13}C$ NMR spectrum of **Mal-Ox-OAc**. | Page S-19 |
| Figure S18 | UHPLC chromatograms of the purified target compounds. | Page S-20 |
| Table S6 | HPLC parameters for SEC-ICP-MS measurements | Page S-21 |
| Table S7 | ICP-MS parameters for SEC-ICP-MS measurements | Page S-21 |

**A**

Hydrolyzed product:
calcd. $C_{25}H_{42}N_4O_{15}Pt$ $(M + H)^+$ = 834.24; found = 834.4

Product:
calcd. $C_{25}H_{40}N_4O_{14}Pt$ $(M + H)^+$ = 816.23; found = 816.3

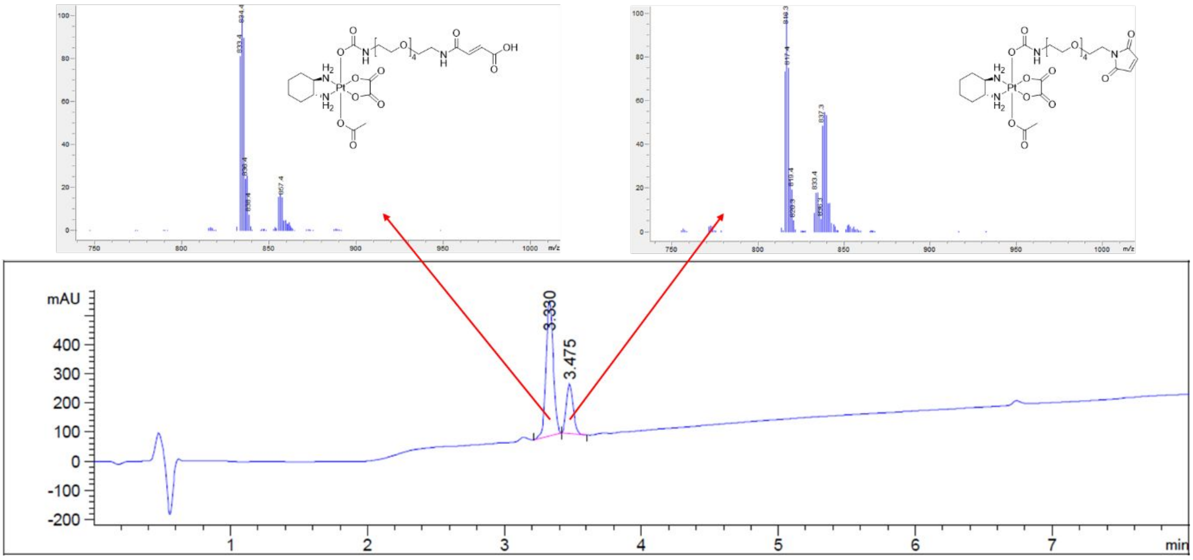


**B**

Hydrolyzed product:
calcd. $C_{27}H_{39}N_5O_{14}Pt$ $(M + H)^+$ = 853.22; found = 853.4

Product:
calcd. $C_{28}H_{41}N_5O_{15}PtS$ $(M + H)^+$ = 915.20; found = 915.4

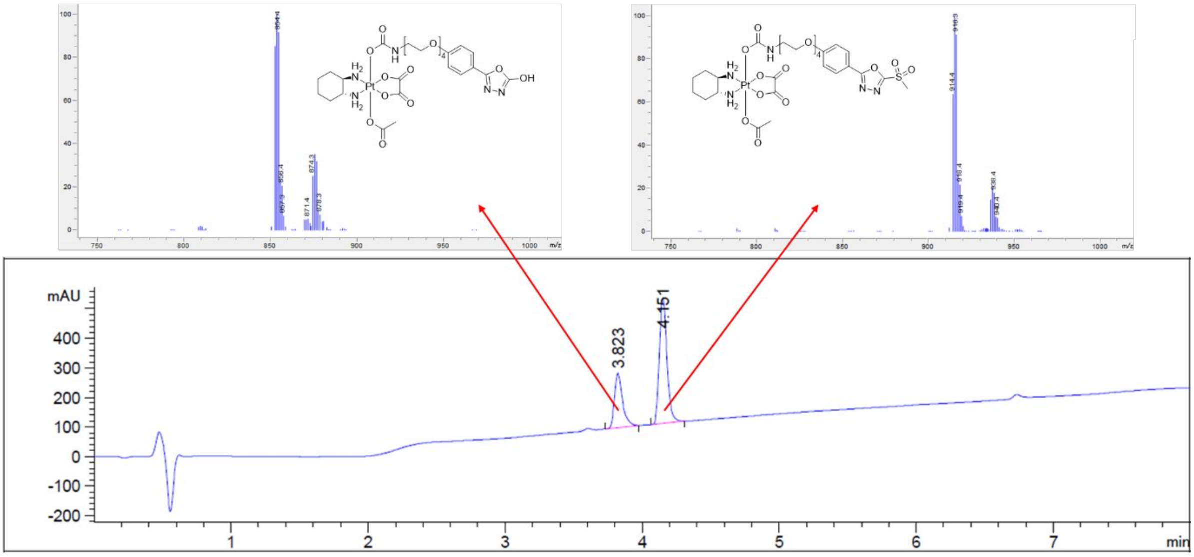

**C**

Product:
calcd. $C_{32}H_{46}F_2N_4O_{15}PtS$ $(M + H)^+$ = 992.24; found = 992.4

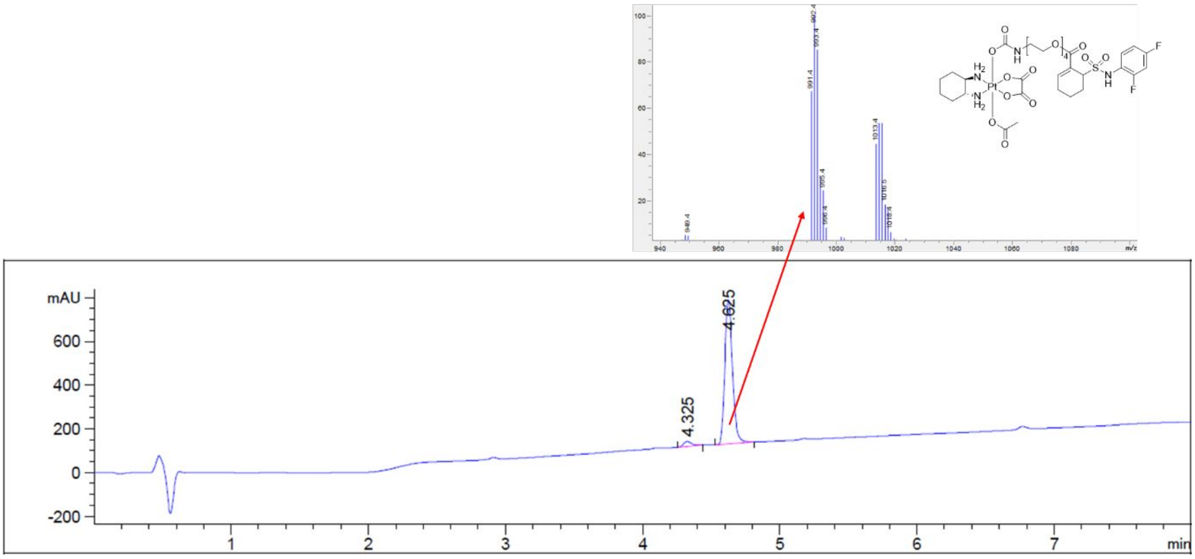

Figure S1: HPLC-MS chromatograms of the samples after the stability measurements (~27 h incubation) in Figure 1. The mass spectra of the respective peaks are indicated with a red arrow. **A**) **Mal-Ox-OAc** elutes at 3.475 min and the maleamic acid hydrolysis product at 3.330 min. **B**) **PODS-Ox-OAc** elutes at 4.151 min and the oxadiazol-2-ol hydrolysis product at 3.823 min. **C**) **DFSA-Ox-OAc** elutes at 4.625 min.

A

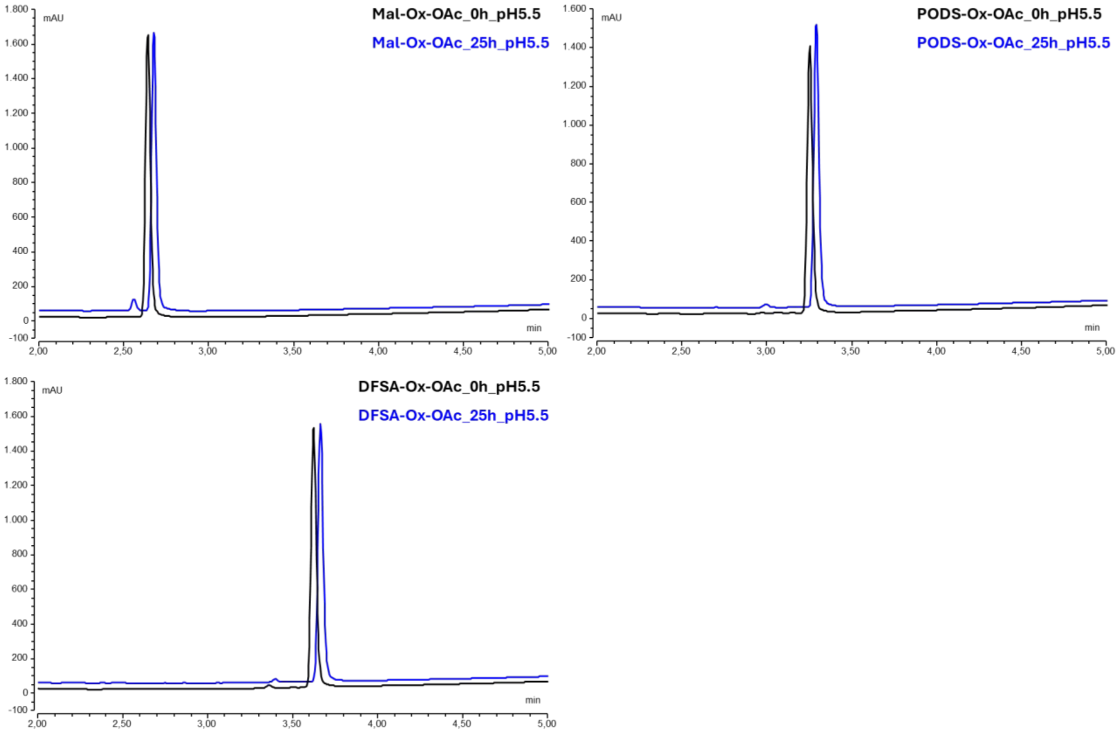


B

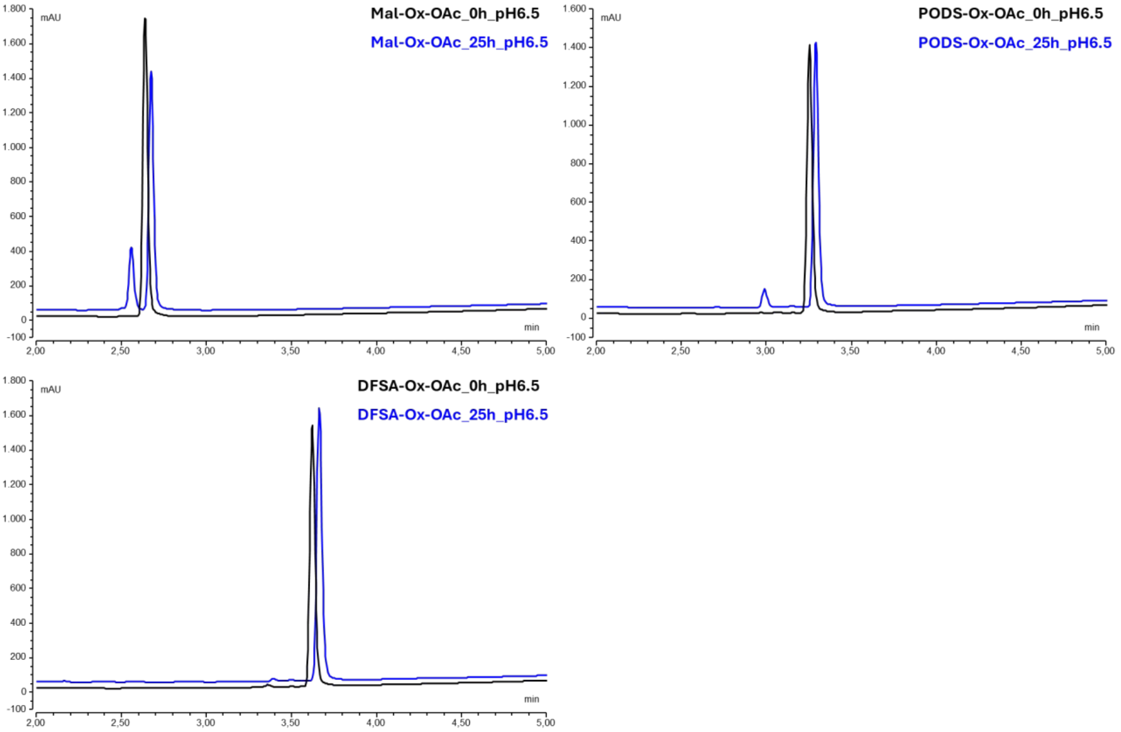


Figure S2: Stability measurements of 0.5 mM **Mal-Ox-OAc**, **PODS-Ox-OAc** and **DFSA-Ox-OAc** at 20°C over 25 h in different pH conditions: **A**) 100 mM PB at pH 5.5, **B**) 100 mM PB at pH 6.5. **Mal-Ox-OAc** elutes at 2.643 min and the maleamic acid hydrolysis product at 2.527 min. **PODS-Ox-OAc** elutes at 3.257 min and the oxadiazol-2-ol hydrolysis product at 2.957 min. **DFSA-Ox-OAc** elutes at 3.623 min. Relative peak areas of all compounds after 25 h of incubation are listed in Table S1.

**C**

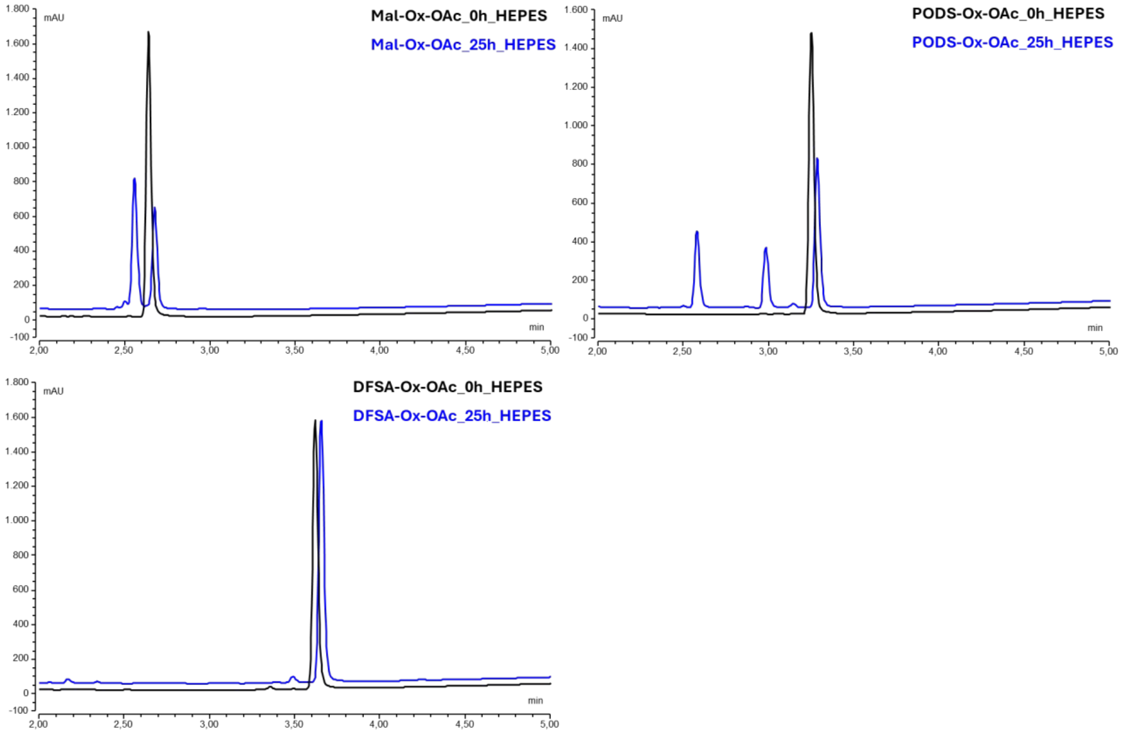


**D**

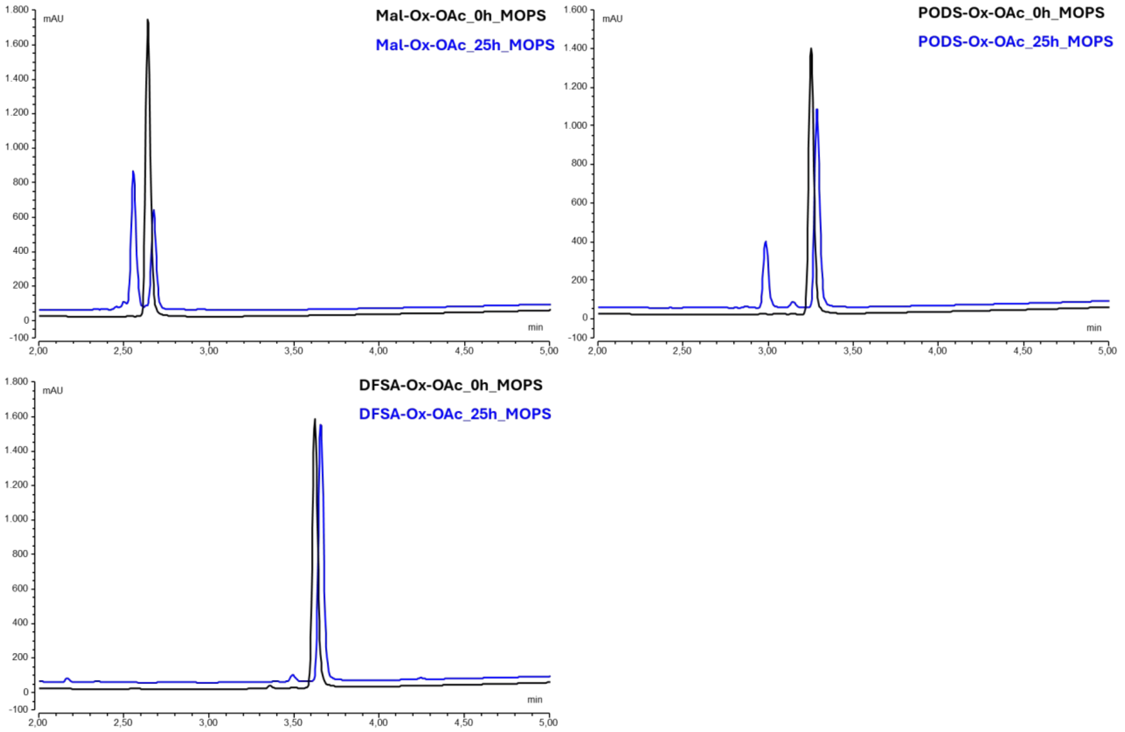


Figure S3 (continued): Stability measurements of 0.5 mM **Mal-Ox-OAc**, **PODS-Ox-OAc** and **DFSA-Ox-OAc** at 20°C over 25 h in different buffer conditions: **C**) 20 mM HEPES at pH 7.4 and **D**) 20 mM MOPS at pH 7.4. **Mal-Ox-OAc** elutes at 2.643 min and the maleamic acid hydrolysis product at 2.527 min. **PODS-Ox-OAc** elutes at 3.257 min and the oxadiazol-2-ol hydrolysis product at 2.957 min. For **PODS-Ox-OAc**, in HEPES buffer an additional peak at 2.547 min was observed after 25 h, which was identified *via* HPLC-MS as the HEPES substitution product (see Figure S3). **DFSA-Ox-OAc** elutes at 3.623 min. Relative peak areas of all compounds after 25 h of incubation are listed in Table S1.

Table S1: **Mal-Ox-OAc**, **PODS-Ox-OAc** and **DFSA-Ox-OAc** after 25 h incubation at 0.5 mM and 20°C in different buffer systems.

| Compound | Intact compound after 25 h at 0.5 mM and 20°C | | | |
|---|---|---|---|---|
| | 100 mM PB, pH = 5.5 | 100 mM PB, pH = 6.5 | 20 mM HEPES, pH = 7.4 | 20 mM MOPS, pH = 7.4 |
| **Mal-Ox-OAc** | 96% | 80% | 56% | 58% |
| **PODS-Ox-OAc** | 99% | 94% | 54% * | 76% |
| **DFSA-Ox-OAc** | stable | stable | stable | stable |

The percentages represent the relative peak areas of the hydrolyzed and intact compound peaks in the chromatograms depicted in Figure S2 (blue traces). *For **PODS-Ox-OAc** in HEPES buffer, the substitution product (see Figure S3) has a relative peak area of 25%.

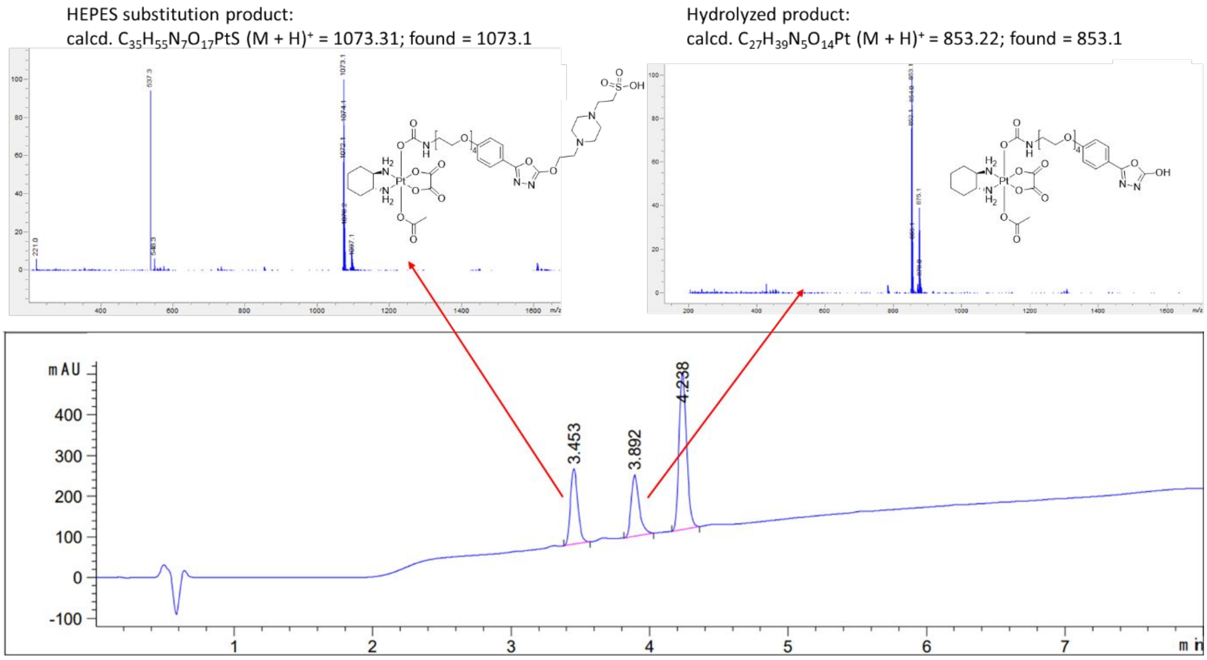


Figure S4: HPLC-MS chromatogram of **PODS-Ox-OAc** after the stability measurement in 20 mM HEPES buffer at pH 7.4 and 20°C (~25 h incubation) in Figure S2C. The mass spectra of the respective peaks are indicated with a red arrow. The peak at 4.238 min corresponds to the intact complex (see Figure S1B). The oxadiazol-2-ol hydrolysis product elutes at 3.823 min and the HEPES substitution product elutes at 3.453 min.

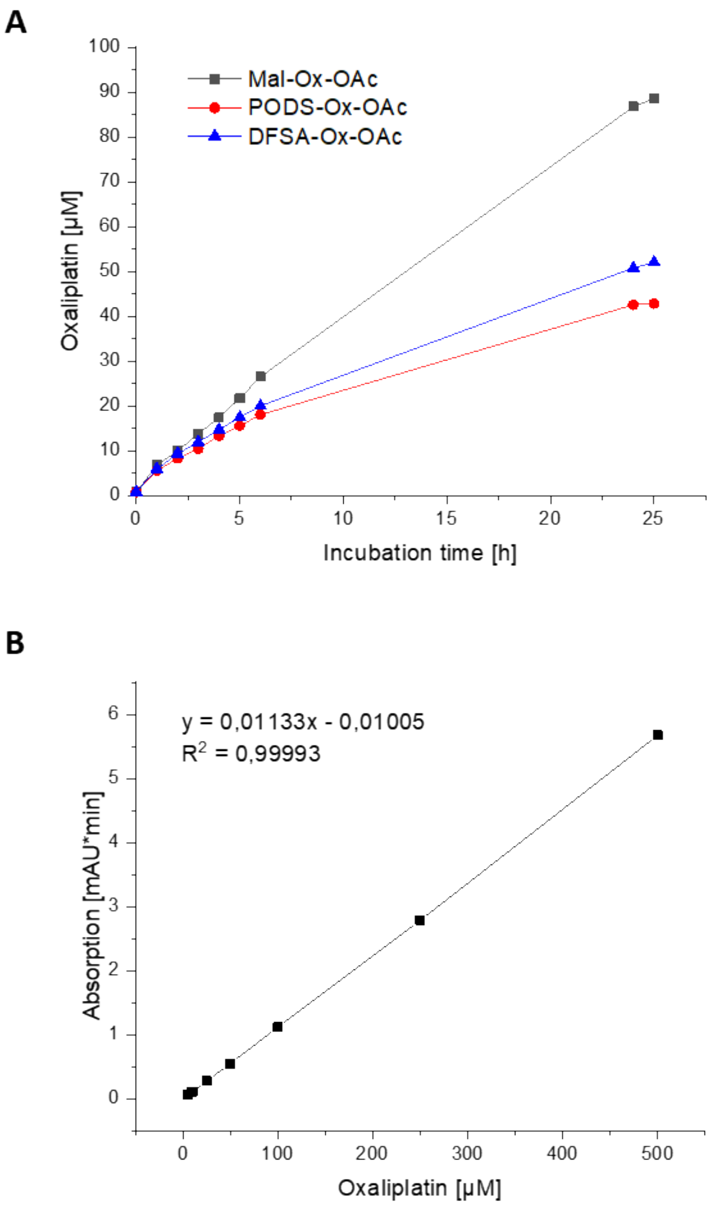


Figure S5: **A**) Reduction behavior of 0.5 mM **Mal-Ox-OAc**, **PODS-Ox-OAc** and **DFSA-Ox-OAc** in 150 mM PB at 20°C and pH 7.4 over 25 h in the presence of 30 eq. AA. The release of oxaliplatin was measured instead of the decrease of the parental compound peak because of the simultaneously occurring ligand hydrolysis. **B**) Calibration curve for the quantification of oxaliplatin between 5–500 µM. Oxaliplatin peaks were integrated at λ = 230 nm.

Table S2: Absorption values of different concentrations of oxaliplatin in 150 mM PB at pH 7.4, measured at 20°C at 0 and 24 h. Oxaliplatin peaks were integrated at λ = 230 nm.

| **Absorption of oxaliplatin in PB** | | | |
|---|---|---|---|
| Conc. [µM] | Abs. at 0 h [mAU*min] | Abs. at 24 h [mAU*min] | Ratio 24/0 h |
| 10 | 0,1167 | 0,1008 | 86% |
| 25 | 0,2819 | 0,2386 | 85% |
| 50 | 0,5620 | 0,4761 | 85% |
| 100 | 1,1249 | 0,9459 | 84% |
| 250 | 2,8374 | 2,3891 | 84% |

Table S3: Percentages of $Pt^{195}$ detected in the high-molecular weight fraction (HMWF; 0-5 min) and low-molecular weight fraction (LMWF; >5 min) of SEC-ICP-MS measurements of **Mal-Ox-OAc**, **PODS-Ox-OAc** and **DFSA-Ox-OAc**.

| **SEC-ICP-MS measurements in mouse serum – 1 h intervals** | | | | | | |
|---|---|---|---|---|---|---|
| Incubation time | Mal-Ox-OAc | | PODS-Ox-OAc | | DFSA-Ox-OAc | |
| | HMWF | LMWF | HMWF | LMWF | HMWF | LMWF |
| 0 h | 34% | 66% | 30% | 70% | 1% | 99% |
| 1 h | 75% | 25% | 82% | 18% | 65% | 35% |
| 2 h | 74% | 26% | 80% | 20% | 78% | 22% |
| 3 h | 74% | 26% | 80% | 20% | 82% | 18% |
| 4 h | 74% | 26% | 79% | 21% | 82% | 18% |
| 24 h | 75% | 25% | 80% | 20% | 70% | 30% |
| **SEC-ICP-MS measurements in mouse serum – 10 min intervals** | | | | | | |
| Incubation time | Mal-Ox-OAc | | PODS-Ox-OAc | | DFSA-Ox-OAc | |
| | HMWF | LMWF | HMWF | LMWF | HMWF | LMWF |
| 0 min | 36% | 64% | 34% | 66% | 2% | 98% |
| 10 min | 58% | 42% | 59% | 41% | 18% | 82% |
| 20 min | 68% | 32% | 71% | 29% | 32% | 68% |
| 30 min | 74% | 26% | 78% | 22% | 43% | 57% |
| 40 min | 78% | 22% | 83% | 17% | 52% | 48% |
| 50 min | 79% | 21% | 85% | 15% | 58% | 42% |
| 60 min | 80% | 20% | 87% | 13% | 63% | 37% |

The compounds were incubated at 100 µM and 37°C in mouse serum, buffered with 150 mM phosphate to pH 7.4. All compounds were measured every hour for 4 h and again after 24 h. **Mal-Ox-OAc**, **PODS-Ox-OAc** and **DFSA-Ox-OAc** were additionally measured in a separate experiment every 10 min for 1 h, using the same conditions.

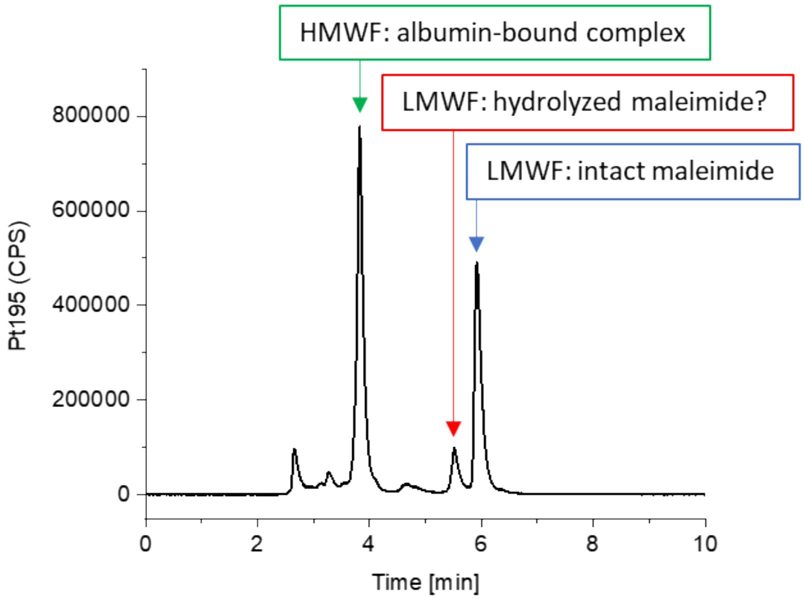


Figure S6: $Pt^{195}$ trace of the **Mal-Ox-OAc** 10 min timepoint from the SEC-ICP-MS measurement depicted in Figure 2D.

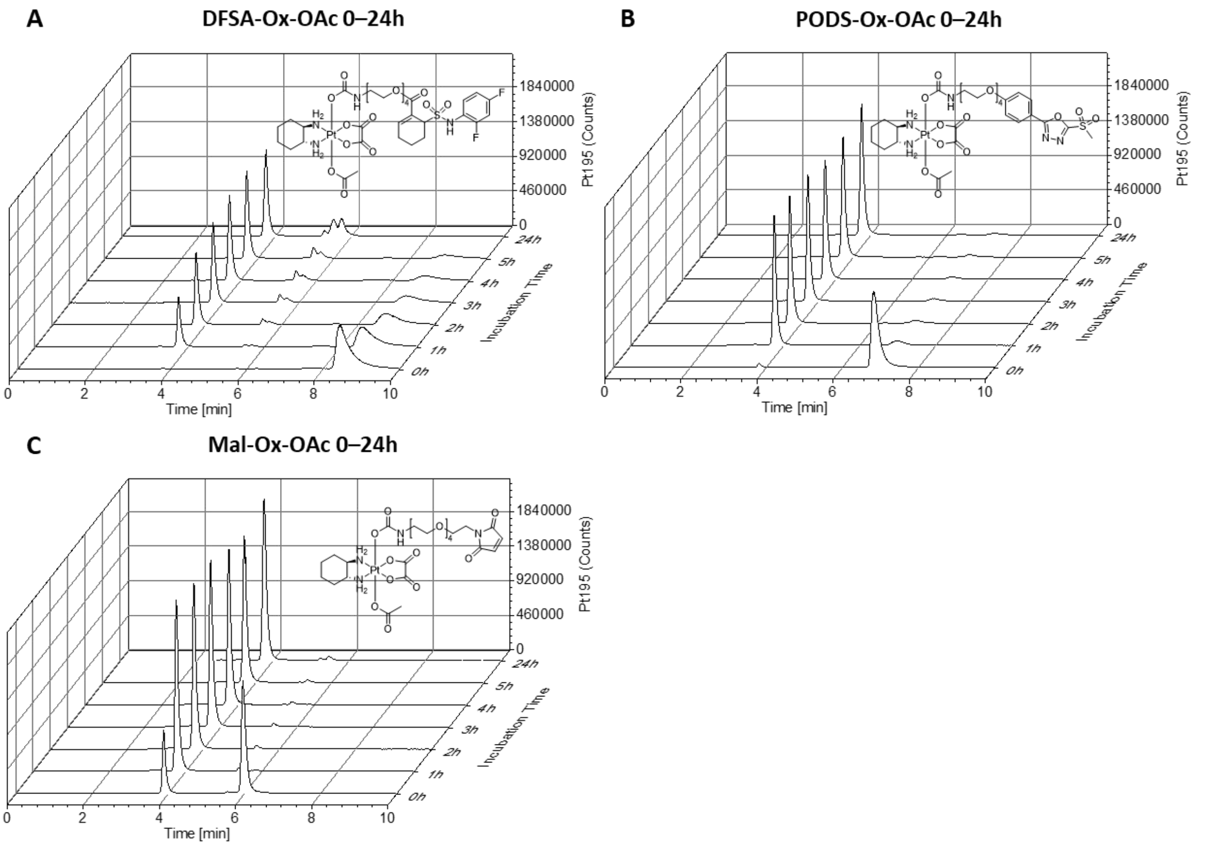


Figure S7: Pt$^{195}$ traces of SEC-ICP-MS measurements. The target compounds were incubated at 100 µM in human serum buffered with 150 mM phosphate at 37°C and pH 7.4. The samples containing **A**) **DFSA-Ox-OAc, B**) **PODS-Ox-OAc** or **C**) **Mal-Ox-OAc** were measured every hour for 5 h and again after 24 h.

Table S4: Percentages of Pt$^{195}$ detected in the high-molecular weight fraction (HMWF; 0-5 min) and low-molecular weight fraction (LMWF; >5 min) of SEC-ICP-MS measurements of **Mal-Ox-OAc**, **PODS-Ox-OAc** and **DFSA-Ox-OAc**.

| **SEC-ICP-MS measurements in human serum – 1 h intervals** | | | | | | |
|---|---|---|---|---|---|---|
| Incubation time | Mal-Ox-OAc | | PODS-Ox-OAc | | DFSA-Ox-OAc | |
| | HMWF | LMWF | HMWF | LMWF | HMWF | LMWF |
| 0 h | 29% | 71% | 2% | 98% | 0% | 100% |
| 1 h | 94% | 6% | 87% | 13% | 34% | 66% |
| 2 h | 94% | 6% | 91% | 9% | 50% | 50% |
| 3 h | 94% | 6% | 92% | 8% | 59% | 41% |
| 4 h | 94% | 6% | 92% | 8% | 63% | 37% |
| 5 h | 94% | 6% | 92% | 8% | 66% | 34% |
| 24 h | 93% | 7% | 92% | 8% | 59% | 41% |

The compounds were incubated at 100 µM and 37°C in human serum, buffered with 150 mM phosphate to pH 7.4. All compounds were measured every hour for 5 h and again after 24 h.

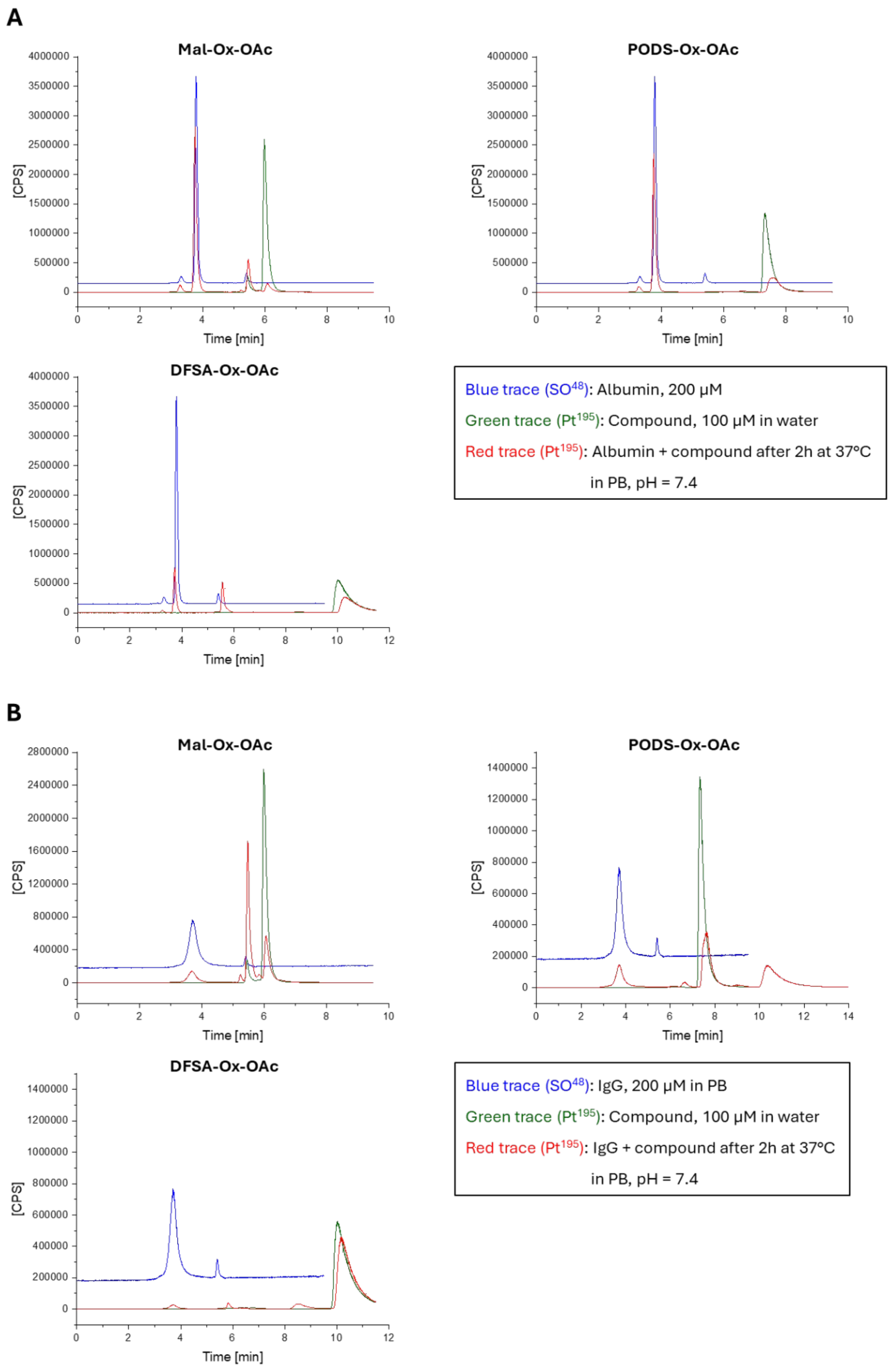


Figure S8: Incubation studies of **Mal-Ox-OAc**, **PODS-Ox-OAc** and **DFSA-Ox-OAc** with the human serum proteins **A**) albumin and **B**) IgG. Depicted are the overlaid SEC-ICP-MS chromatograms of the protein $SO^{48}$ trace (blue, 200 µM in 100 mM PB buffer at pH 7.4), the pure compound $Pt^{195}$ trace (green, 100 µM in Milli-Q water) and the $Pt^{195}$ trace after incubation of protein and compound for 2 h at 37°C (red).

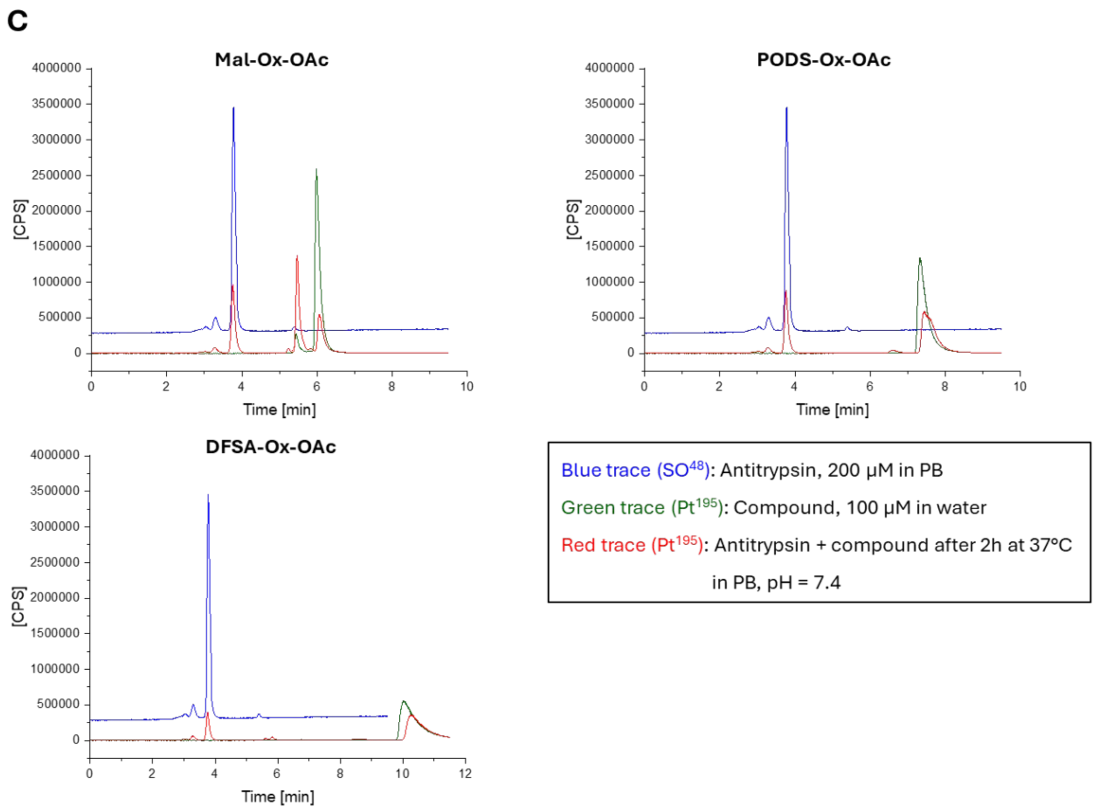


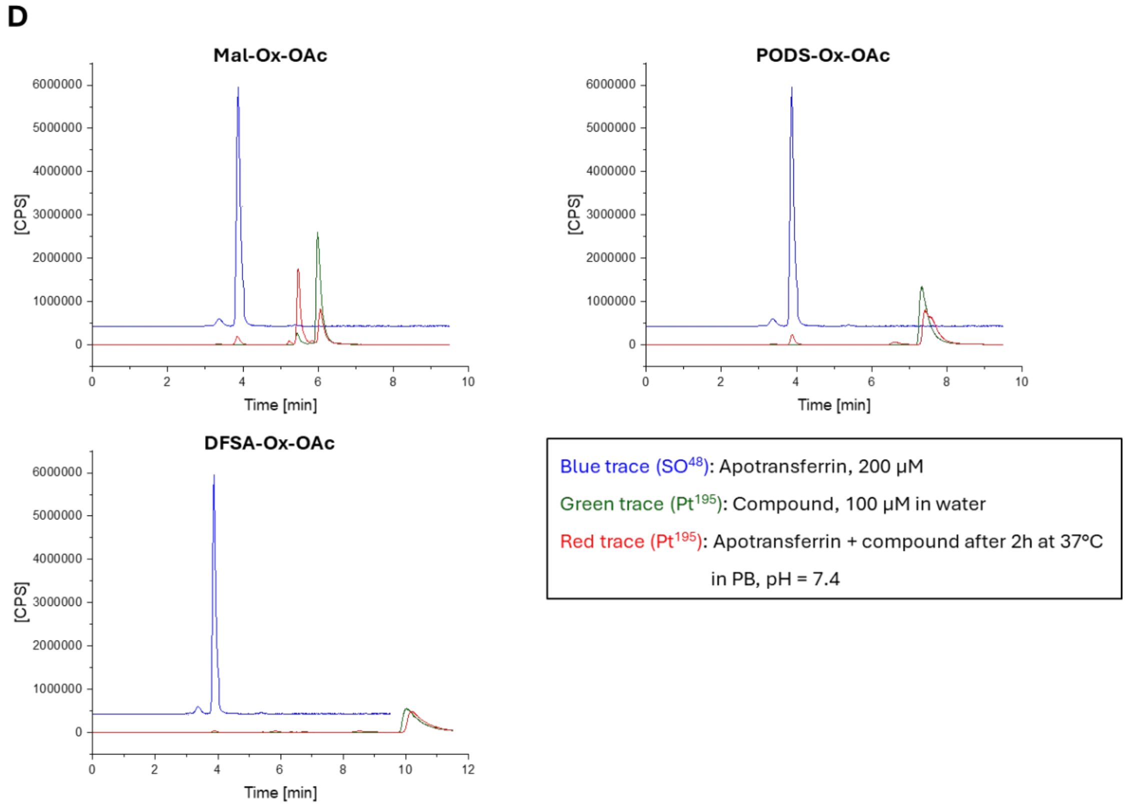


Figure S9 (continued): Incubation studies of **Mal-Ox-OAc**, **PODS-Ox-OAc** and **DFSA-Ox-OAc** with the human serum proteins **C**) α1-antitrypsin and **D**) apotransferrin. Depicted are the overlaid SEC-ICP-MS chromatograms of the protein $SO^{48}$ trace (blue, 200 µM in 100 mM PB buffer at pH 7.4), the pure compound $Pt^{195}$ trace (green, 100 µM in Milli-Q water) and the $Pt^{195}$ trace after incubation of protein and compound for 2 h at 37°C (red).

Table S5: Pharmacokinetic parameters of the different compounds based on serum levels.

| | PODS-Ox-OAc | Mal-Ox-OAc | DFSA-Ox-OAc | Oxaliplatin |
|---|---|---|---|---|
| $AUC_{Pt\_0\text{-}24}$ [mg·h/kg] | 461 | 369 | 35.1 | 26.6 |
| $AUC_{Pt\_0\text{-}\infty}$ [mg·h/kg] | 638 | 496 | 64.5 | 37.5 |
| $C_{max}$ [mg/kg] | 52.5 | 35.6 | 4.61 | 6.17 |
| CL [kg/h/kg] | 0.00693 | 0.00892 | 0.0685 | 0.118 |
| $V_z$ [kg/kg] | 0.136 | 0.157 | 2.17 | 2.42 |
| Half-life [h] | 13.6 | 12.2 | 21.9 | 14.3 |

Non-compartmental analysis was based on a dose of 4.42 mg/kg Pt; $AUC_{Pt\_0\text{-}24}$: area-under-the-concentration-time-curve from time of dosing to $t_{last}$=24 h; $AUC_{Pt\_0\text{-}\infty}$: AUC from time of dosing extrapolated to infinity based on the last predicted concentration (of note, percentage of $AUC_{Pt\_0\text{-}\infty}$ extrapolated from $t_{last}$ to infinity: Mal-Ox-OAc/PODS-Ox-OAc/DFSA-Ox-OAc/Oxaliplatin: 27.7/25.5/45.6/ 29.0%); $C_{max}$: maximum observed concentration, measured at $t_{max}$=0.08 h; CL: clearance: total body clearance; $V_z$: volume of distribution based on the terminal phase; Half-life refers to terminal half-life.

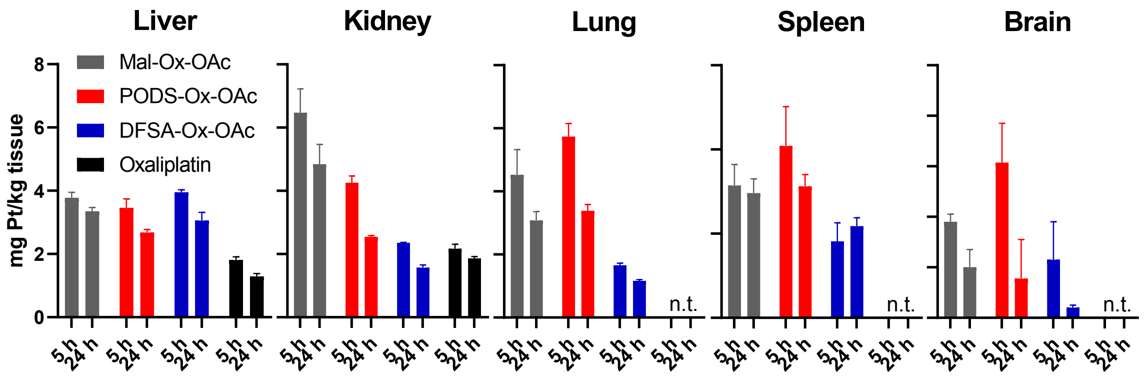


Figure S10: Organ distribution of the platinum complexes in CT-26-bearing Balb/c mice. Animals were treated once i.v. with concentrations equimolar to 9 mg/kg oxaliplatin. Organ samples were collected after 5 h and 24 h. Platinum levels of all samples were measured via ICP-MS. Data given are means ± SEM. n.t. = not tested.

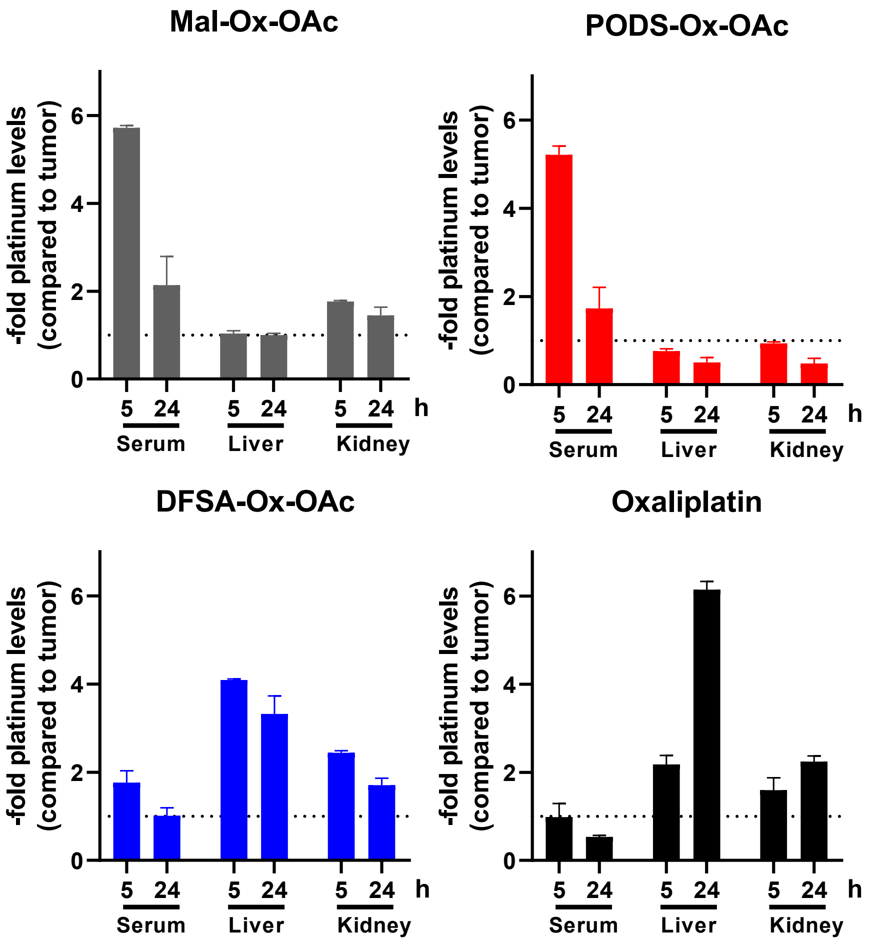


Figure S11: Ratio of organ to tumor distribution of the platinum complexes in CT-26-bearing Balb/c mice. For each indicated compound, platinum levels in the respective organs are given normalized to the corresponding tumor tissue. Dashed line indicates 1-fold (compared to platinum levels in tumor). Data given are means ± SEM.

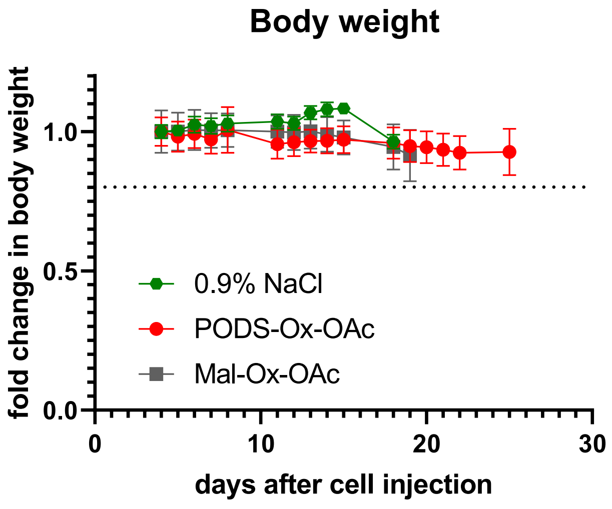


Figure S12: Changes in body weight during therapy. First treatment day is set to 1. CT26-bearing Balb/c mice (male) were treated with the indicated drugs equimolar to 9 mg/kg oxaliplatin i.v. on day 4, 8, 12 after cell injection. Data shown until first animal of the respective treatment groups had to be sacrificed. Data are means ± SEM.

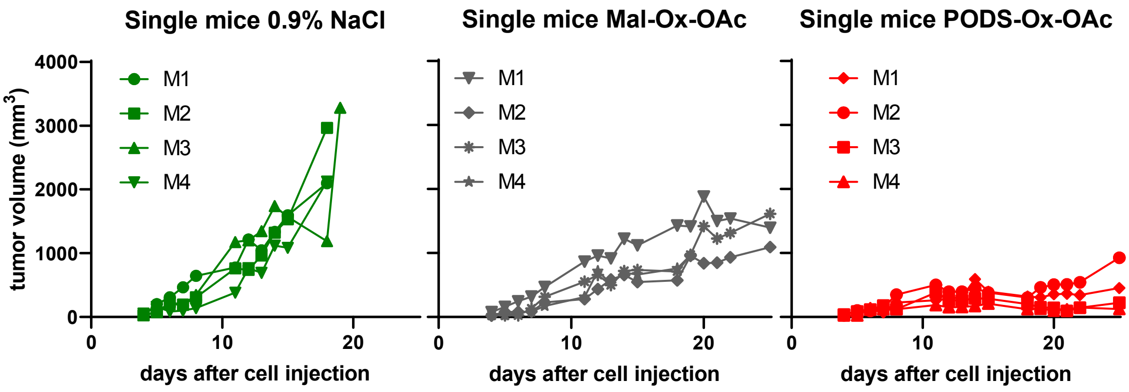


Figure S13: Tumor volume of the individual CT26-bearing mice after i.v. treatment of the indicated drugs (equimolar to 9 mg/kg oxaliplatin, on days 4, 8, and 12 post-cell injection). Endpoint of the experiments was overall survival.

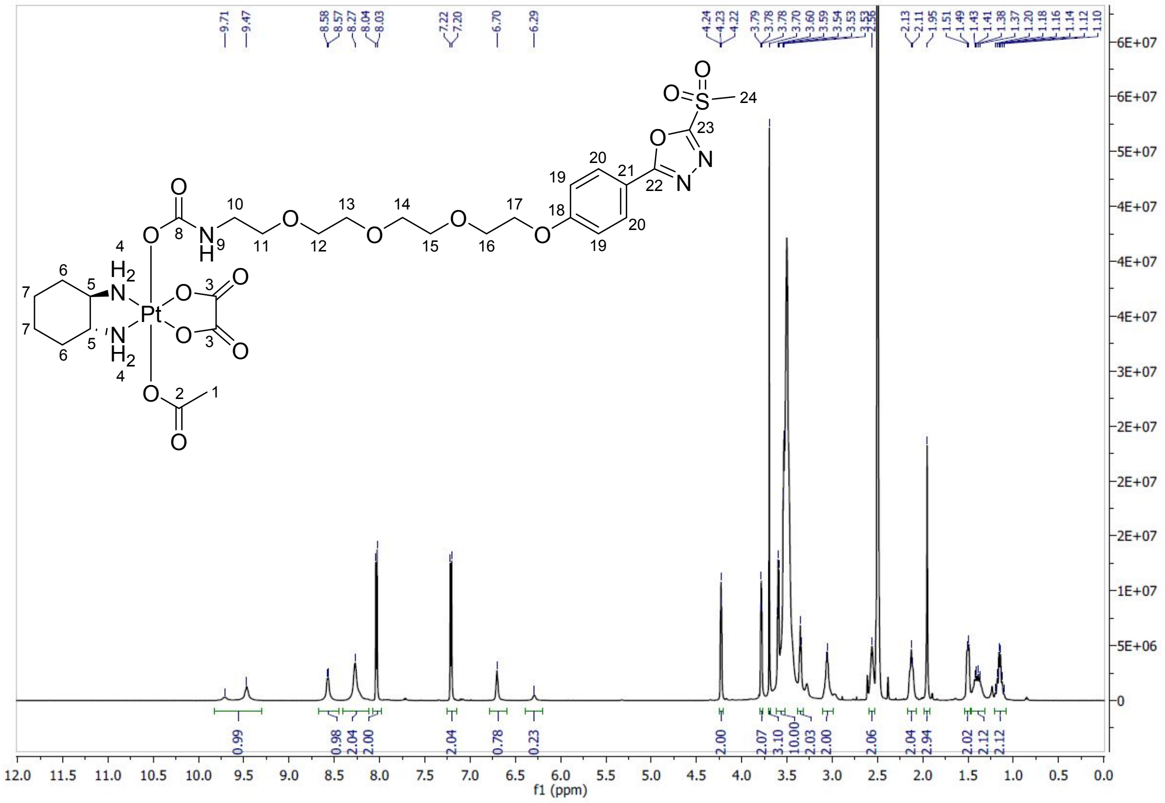


Figure S14: Structure and $^{1}H$ NMR spectrum of **PODS-Ox-OAc**.

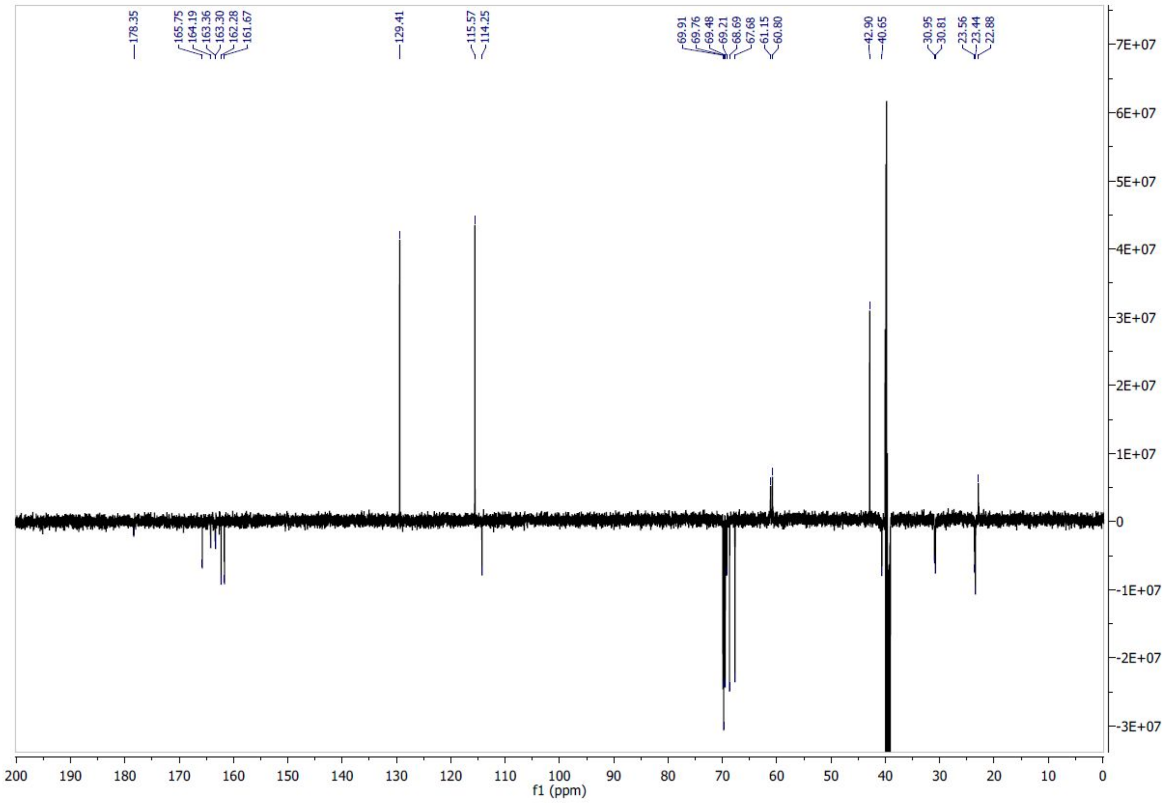


Figure S15: $^{13}C$ NMR spectrum of **PODS-Ox-OAc**.

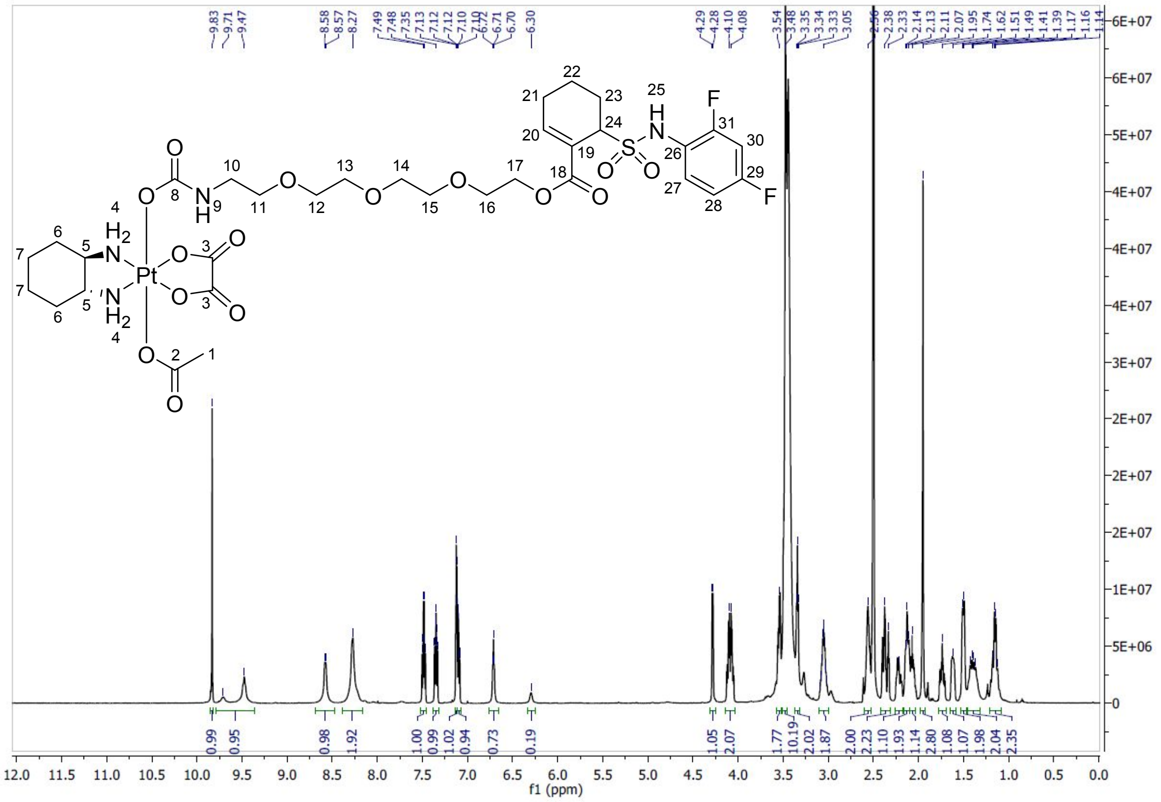

Figure S16: Structure and $^{1}H$ NMR spectrum of **DFSA-Ox-OAc**.

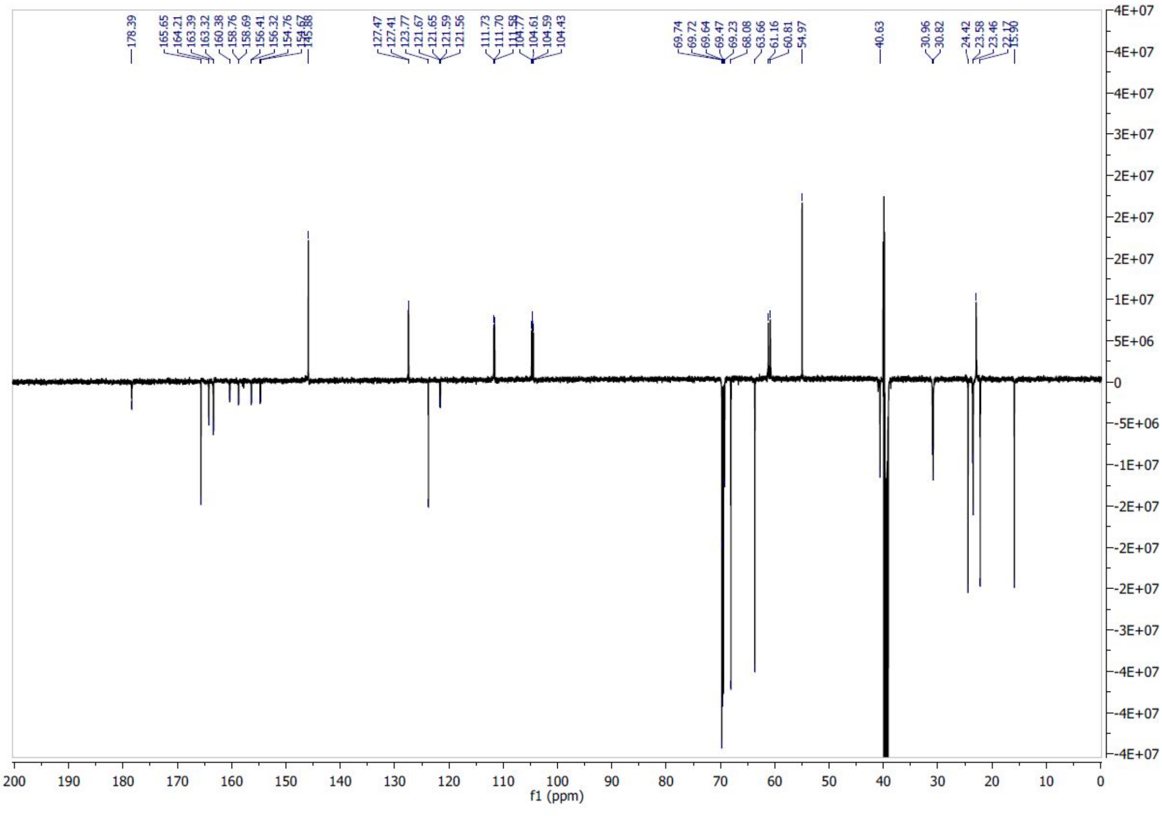

Figure S17: $^{13}C$ NMR spectrum of **DFSA-Ox-OAc**.

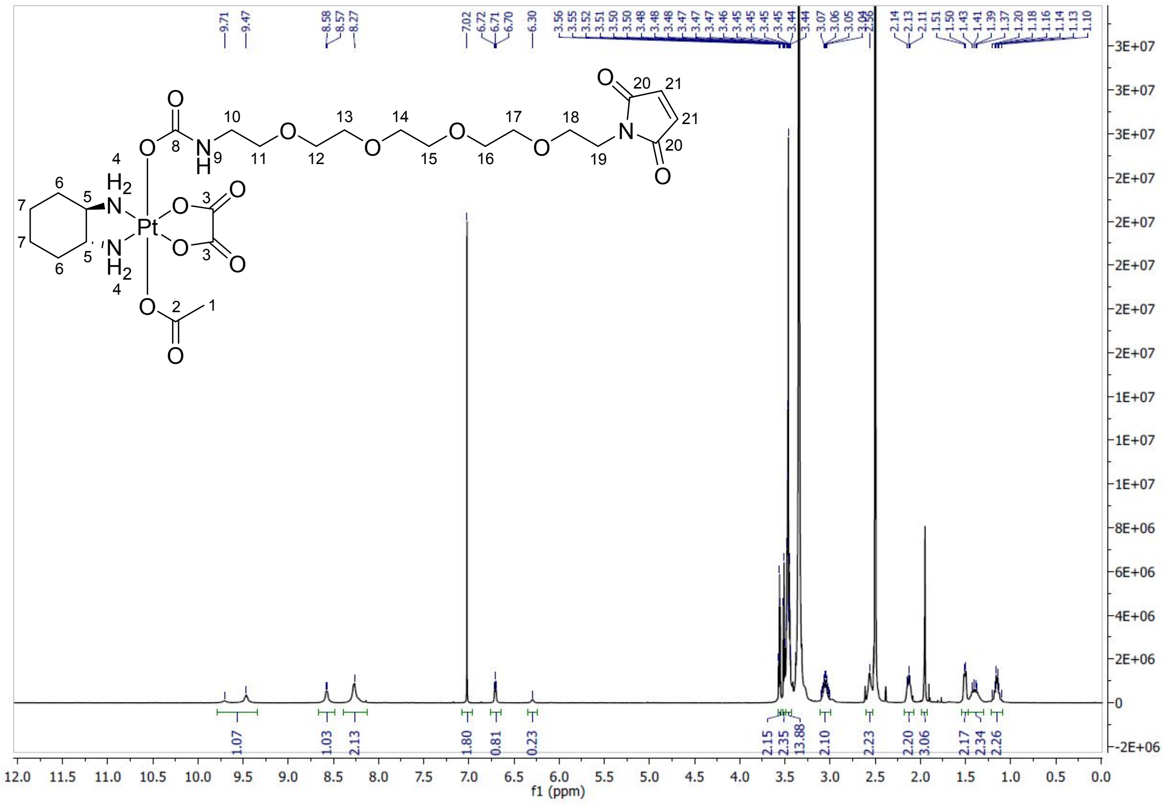

Figure S18: Structure and $^{1}H$ NMR spectrum of **Mal-Ox-OAc**.

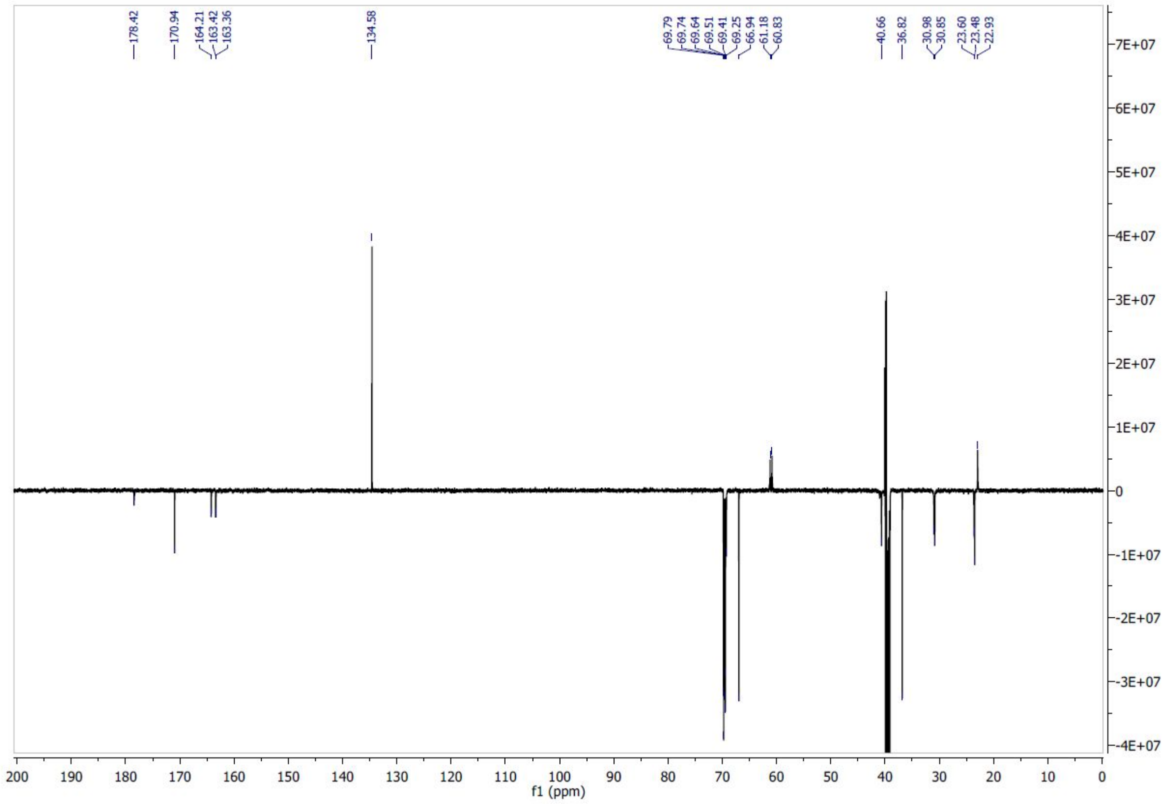

Figure S19: $^{13}C$ NMR spectrum of **Mal-Ox-OAc**.

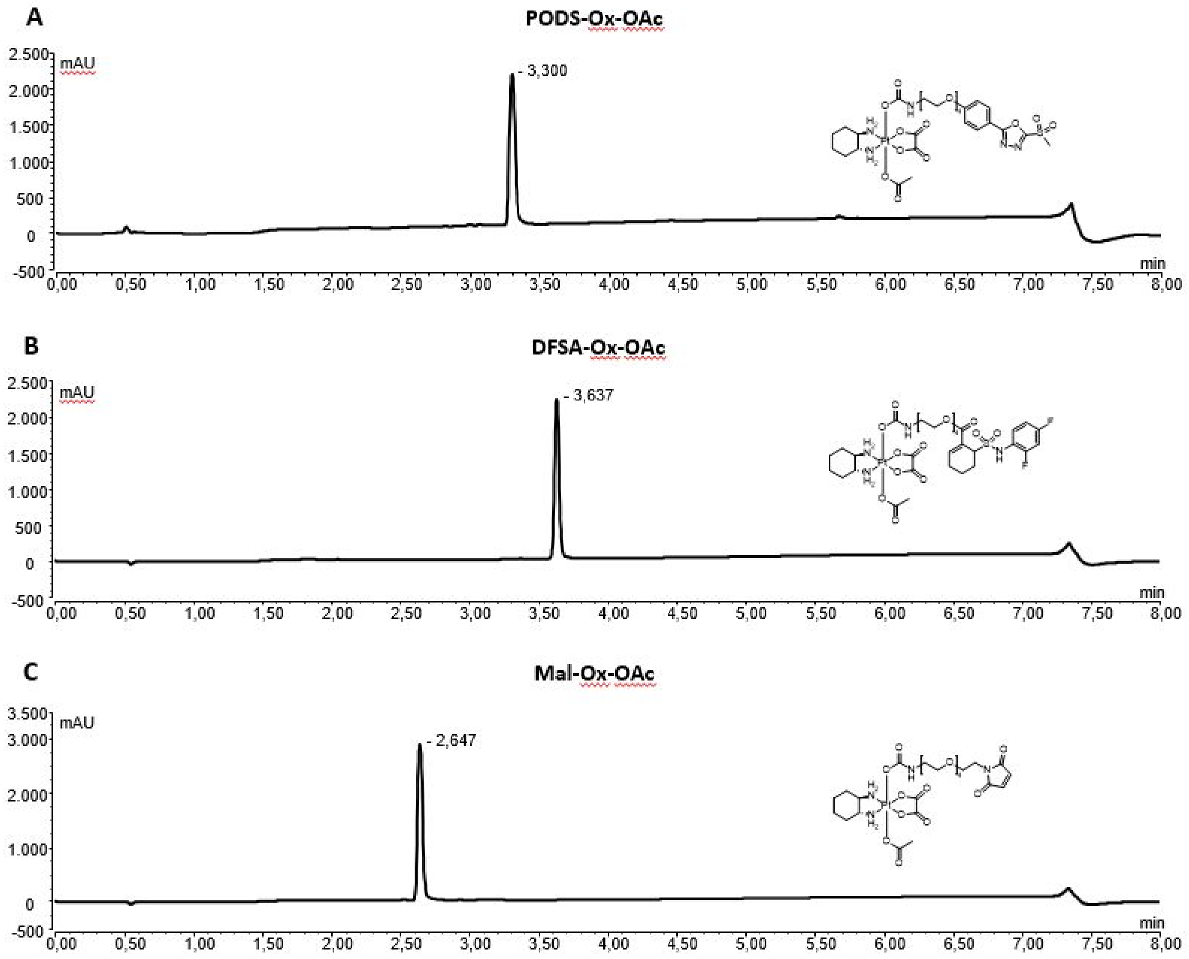


Figure S20: The purified complexes **A**) **PODS-Ox-OAc**, **B**) **DFSA-Ox-OAc** and **C**) **Mal-Ox-OAc** were analyzed with analytical UHPLC at 220 nm. **PODS-Ox-OAc** elutes at 3.30 min, **DFSA-Ox-OAc** at 3.64 min and **Mal-Ox-OAc** at 2.65 min. UHPLC Conditions: Milli-Q water (mobile phase A) and acetonitrile (mobile phase B), both containing 0.1% TFA, were used as eluents. The flow rate was consistent at 0.6 mL/min for all measurements using the following gradient: 0–0.5 min A 95:5 B, 0.5–6-0 min linear gradient to A 5:95 B, 6.0–7.0 min A 5:95 B, 7.0–7.1 min linear gradient to A 95:5 B, 7.1–8.0 min A 95:5 B.

Table S6: HPLC parameters for SEC-ICP-MS measurements

| | |
|---|---|
| HPLC column | Acquity UPLC® BEH 200 Å 1.7 µm, 4.6 x 150 mm |
| Eluent | 50 mM $CH_3COONH_4$, pH = 6.8 |
| Flow rate | 0.4 mL/min |
| Injection volume | 0.5 µL |
| Temperature autosampler | 37°C |
| Temperature column oven | 37°C |

Table S7: ICP-MS parameters for SEC-ICP-MS measurements

| | |
|---|---|
| Nebulizer | Quartz |
| Spray chamber | Scott type |
| Nebulizer gas flow | 1.08 L/min |
| Aux. gas flow | 0.9 L/min |
| Plasma gas flow | 15 L/min |
| Reaction gas (oxygen) | 30% |
| ICP RF power | 1550 W |
| m/z measured | 195, 48 |
